# Structural and Optical Properties of Pure and Sulfur-Doped Silicate–Phosphate Glass

**DOI:** 10.3390/molecules26113263

**Published:** 2021-05-28

**Authors:** Justyna Sułowska, Dominika Madej, Bartłomiej Pokrzywka, Magdalena Szumera, Andrzej Kruk

**Affiliations:** 1 Department of Ceramics and Refractories, Faculty of Materials Science and Ceramics, AGH University of Science and Technology, A. Mickiewicza 30, 30-059 Krakow, Poland; dmadej@agh.edu.pl (D.M.); mszumera@agh.edu.pl (M.S.); 2 Institute of Physics, Pedagogical University, Podchora̧żych 2, 30-084 Krakow, Poland; bartlomiej.pokrzywka@up.krakow.pl; 3 Institute of Technology, Pedagogical University, Podchorążych 2, 30-084 Krakow, Poland

**Keywords:** silicate–phosphate glass, sulfur, luminescence, IR spectroscopy

## Abstract

A series of silicate–phosphate glass materials from the SiO_2_-P_2_O_5_-K_2_O-MgO system (pure and doped with sulfur ions) were synthesized by melting raw material mixtures that contained activated carbon as a reducer. The bulk composition of glass was confirmed with X-ray fluorescence spectroscopy. The homogeneity of the glass was confirmed through elemental mapping at the microstructural level with scanning electron microscopy combined with an analysis of the microregions with energy-dispersive X-ray spectroscopy. The structural and optical properties of the glass were studied by using spectroscopic techniques. The infrared spectroscopy studies that were conducted showed that the addition of sulfur caused changes in the silicate–phosphate networks, as they became more polymerized, which was likely related to the accumulation of potassium near the sulfur ions. By using irradiation with an optical parametric oscillator (OPO) nanosecond laser system operating at the second harmonic wavelength, the glass samples emitted a wide spectrum of luminescence, peaking at about 700 nm when excited by UV light (210–280 nm). The influence of the glass composition and the laser-processing parameters on the emission characteristics is presented and discussed. This work also referred to the density, molar volume, and theoretical optical basicity of pure and sulfur-doped glass.

## 1. Introduction

The element sulfur is characteristically heterovalent, exhibits a great range of oxidation states (−2 to +6), and forms chemical bonds with both more electropositive and more electronegative elements. Under reducing conditions, sulfur behaves as an anion, forming bonds directly with metal cations, whereas under oxidizing conditions, it forms anions with oxygen [1]. A large number of sulfur species have been recognized, including groups containing sulfur of a single valence state, such as sulfate (SO_4_^2−^), sulfite (SO_3_^2−^), dithionate ([O_3_S-SO_3_]^2−^, dithionite ([O_2_S-SO_2_]^2−^, elemental sulfur (S_8_), and sulfide (S^2−^), and those of two or more different valence states, e.g., thiosulfate ([S(O_3_S)^2−^] and polysulfides [(S-(S_n_)-S]^2−^) [1,2,3]. According to the literature [4,5], sulfur is predominantly dissolved in glass as SO_4_^2−^ and S^2−^. Sulfur is one of the elements whose different valence states correlate directly with different coordination environments [6]. The degree of oxidation of sulfur has a significant effect on the processes of melting, formation, and clarification, as well as on the glass structure and properties of the final product, i.e., the viscosity, mechanical properties, and optical properties (light transmittance and color) [7].

The degree of oxidation of sulfur in the structure of glass can be roughly determined based on the glass color that is obtained. The authors of [8] showed that the average oxidation state of sulfur in the structure of glass from the SiO_2_-Al_2_O_3_-Na_2_O-K_2_O-MgO-CaO-Fe_2_O_3_-Cr_2_O_3_-SO_3_ system decreases corresponding to the following progression of colors: colorless (SO_4_^2−^) → light olive → dark olive → light amber → dark amber (S^2−^).

Sulfate can form oxoanionic glass as the only anion, but single-component sulfate glass is not known to vitrify easily [9]. Sulfur is a poorly glass-compatible element. Sulfur can be incorporated into silicate and borosilicate glass structures at up to 1 wt.% of SO_3_ equivalent. More than 1 wt.% SO_3_ causes the formation of a separate water-soluble (yellow) phase that contains alkali sulfates [10].

According to the literature, the solubility of sulfur in the structure of phosphate glass is higher than that in silicate or borosilicate glass [11], but there is evidence of phase separation and crystallization when a large amount of sulfate is added into phosphates [12,13].

The volatilization of SO_2_ gas due to the synthesis of sulfate glass is the largest problem. At the same time, because of this phenomenon, sulfate is used in many glass melts as a fining agent to promote melt homogeneity [14].

Infrared spectroscopy is frequently applied to crystalline sulfides and sulfates to study the vibrational modes of these compounds [15,16,17]. However, only a few studies can be found that report infrared spectra with a focus on sulfur in glass. These are mostly restricted to chalcogenide-based glass with very high sulfur contents or other compositions of technical interest [18,19,20]. This literature review shows that there is a literature gap that would be filled by addressing the subject of infrared spectra focused on sulfur in silicate–phosphate glass. The literature on the infrared spectroscopy of sulfur in silicate glass is scarce, but there are a few reports in the literature on the infrared spectra of sulfur in phosphate glass [21,22,23,24].

Photoluminescence is a very sensitive analytical method. The optical properties of glass and glass ceramics made for different applications depend strongly on the active centers, the surrounding host composition, and their interactions. Static and time-resolved photoluminescence in the UV-VIS range is a very sensitive method for detecting local structures of luminescent ions depending on the surrounding glass and crystal matrix. Luminescent glass and glass ceramics have a large potential for many applications in the field of photonics [25].

According to the literature [26,27,28], the color centers induced in sulfur-doped silica glass stand out due to their unique spectroscopic properties. Gerasimova et al. [26,27,28] assigned the absorption band at 280 nm and the photoluminescence band at 385 nm to S_2_ interstitial molecules, whereas the absorption bands at 237 and 400 nm were attributed to S^2+^, which they called an interstitial molecular ion. In our opinion, this is questionable because it is a radical. To continue the study of the photoluminescence of sulfur-doped silica glass, upon the excitation of the absorption band at 203 nm at a temperature of 10 K, the authors assigned the bands of absorption at 203 nm and luminescence at 420 nm to the interstitial SO_2_ molecules that were weakly bound to the glass network.

Recently, there has been interest in research on luminescent lead sulfide quantum dots in glass enriched in sulfur [29,30,31]. There is a growing interest in families of special glass that involve chalcogenide glass systems, e.g., As–S and Ge-S, due to their promising properties, such as the transmission in the middle and far infrared regions of spectra, lower values of phonon energies, and higher values of refractive indexes compared to SiO_2_ [32,33].

The aim of this study was to determine the structure and optical properties of pure and sulfur-doped silicate–phosphate glass with varying P_2_O_5_ content obtained under reducing conditions. Such glass could find application as glassy carriers of sulfur for soil environments [34,35,36]. Thus, we can try to solve the problem of sulfur deficiency in soil. This study is intended to enable the indirect assessment of the form of sulfur in glass structures.

## 2. Results and Discussion

### 2.1. Compositional Analysis of the Glass

A visual evaluation of the sulfur-doped glass obtained showed that they were dark in color (in a range of brown and red colors) and amorphous. According to the literature, the color of the glass may come from the presence of K_2_S_6_-type connections in the structure, which can give the materials a dark red color [37].

The pure 45Si2P, 43Si5P, and 39Si8P glasses (sulfur-free) were homogenous and transparent, with no signs of crystallization in their appearance. Visual observations indicated a glass–crystalline character of the 36Si11P material.

**Figure 1:**
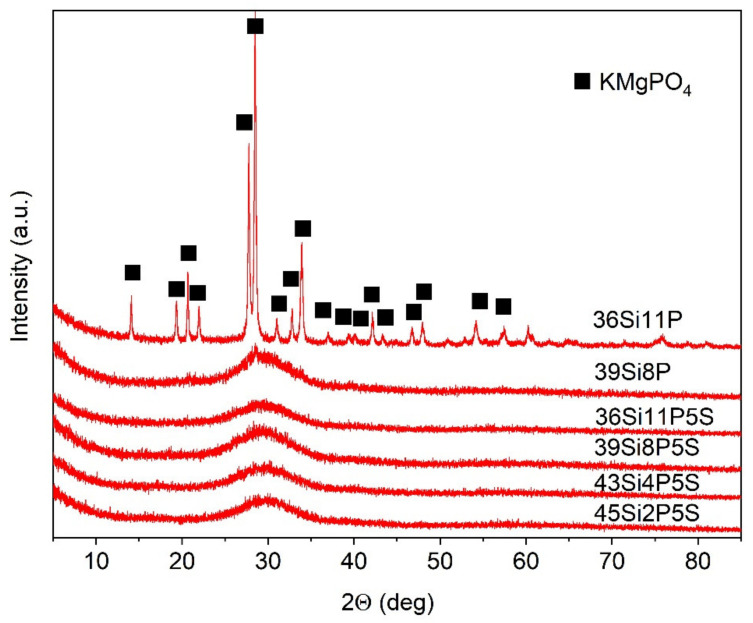
XRD patterns of the studied glass materials.

The X-ray diffraction spectra of the prepared glass samples are shown in Figure 1.

The XRD studies confirmed the amorphous nature of all of the sulfur-doped glass materials (Figure 1), as evidenced by the characteristic broad hump centered at 2θ = 30°. The XRD studies confirmed the crystallization of the 36Si11P melts during their cooling. The phase composition of the 36Si11P material carried out using X’Pert HighScore Plus program indicated the presence of potassium magnesium phosphate KMgPO4 (ICDD: 00-050-0146) in its structure.

Table 1 presents the compositions of the pure and sulfur-doped glass as analyzed with X-ray fluorescence spectrometry (XRF).

The difference between the nominal (5 mol.%) and measured sulfur content was likely due to the evaporation of sulfate during the melting of the glass batches. Additionally, tiny amounts of Al_2_O_3_ (0.648–1.121 mol.%) from the crucible dissolution also affected the glass compositions.

The sulfur content measured in the sulfur-doped glass—expressed as sulfur trioxide (SO_3_)—varied from 1.721 to 4.541 mol.%. The sulfur content measured in the 45Si2P5S glass (1.721 mol.% SO_3_) represented 34.4% of the total quantity of sulfur (5 mol.%) that was introduced into the 45Si2P5S glass batch.

In the case of the 39Si8P5S and 36Si11P5S glass samples, it was possible to retain about 60% of the nominal amount of sulfur that was introduced into the glass batches for these glass samples. The greater content of sulfur introduced into the structures of the 39Si8P5S and 36Si11P5S glass samples could be associated with the higher content of P_2_O_5_ in their structures, despite the content of P_2_O_5_ that was much lower than nominal in the case of the 39Si8P5S glass sample. On the other hand, the largest amount of sulfur was introduced into the structure of the 43Si4P5S glass sample in the amount of approximately 91% of the nominally introduced SO_3_ in its glass batch.

**Figure 2:**
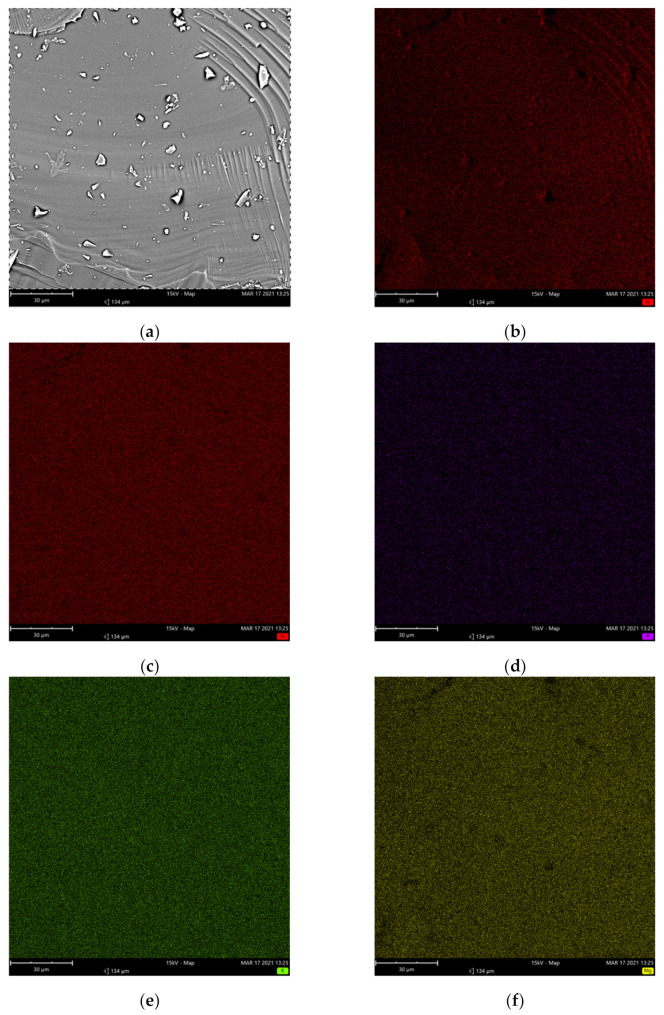
SEM image of the 45Si2P glass sample (backscattered electrons: BSEs) (**a**) and associated EDS elemental maps for (**b**) O, (**c**) Si, (**d**) P, (**e**) K, and (**f**) Mg.

**Figure 3:**
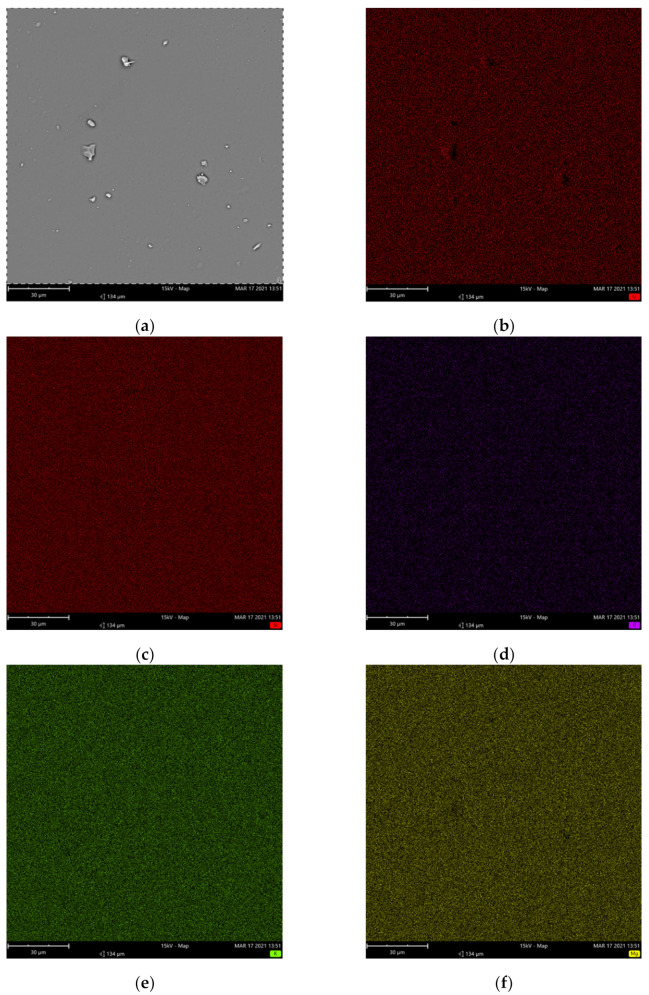

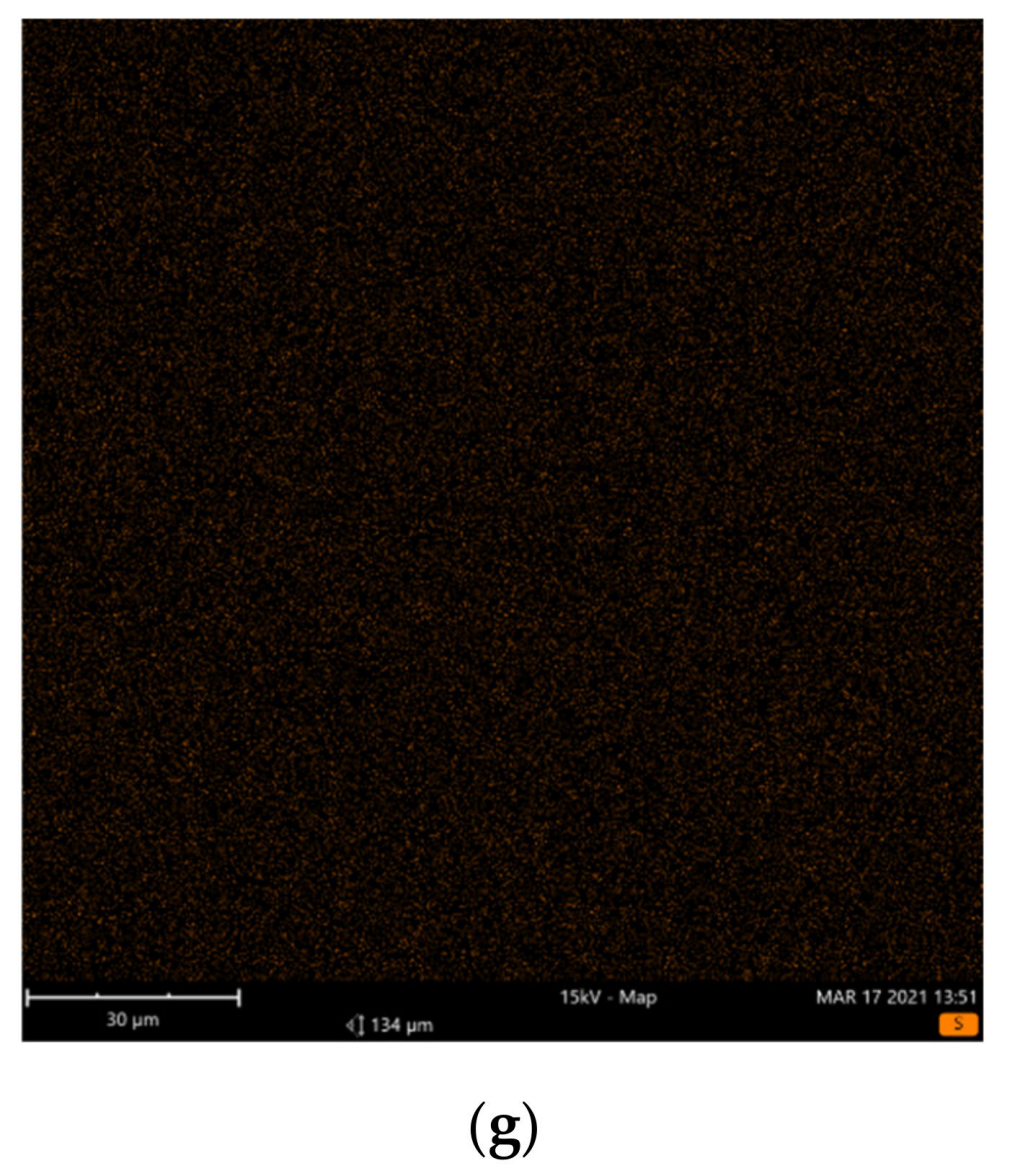
SEM image of the 45Si2P5S glass sample (backscattered electrons: BSEs) (**a**) and associated EDS elemental maps for (**b**) O, (**c**) Si, (**d**) P, (**e**) K, (**f**) Mg, and (**g**) S.

The results of the elemental mapping at the microstructural level with scanning electron microscopy (SEM) and energy-dispersive X-ray spectrometry (EDS) are presented for the 45Si2P (Figure 2) and 45Si2P5S glass samples (Figure 3). The SEM images in Figure 2 and Figure 3 show some features that are due to the preparation of the samples.

It can be observed from the EDS elemental mapping in Figure 2 and Figure 3 that there was a uniform distribution of the elements O, Si, K, Mg, and P for the pure 45Si2P glass sample, in addition to S in the case of the sulfur-doped 45Si2P5S glass sample. Magnesium and potassium were widely spread throughout the glass’ surface. Silicon was more concentrated, but less so than oxygen. The EDS measurement showed that P and S (for sulfur-doped glass) were the least concentrated elements in the analyzed area, and were uniformly present in the glass.

No sulfate layers or holes left by gas bubbles were detected on the surfaces of the sulfur-doped glass [10,38].

### 2.2. Density and Molar Volume

#### 2.2.1. Density

The true density values of all of the obtained materials—one of the most important physical properties for evaluating the compactness of glass structures—are shown in Table 2.

As indicated in Table 2, the average densities of all materials without sulfur are in the range of 2.5918–2.5084 g/cm^3^, while those containing sulfur are in the range of 2.5682–2.4919 g/cm^3^, which means that the value of this parameter decreased due to the presence of sulfur in the materials. It is worth noting that the density of the sulfur-free glass samples is greater than the density of their sulfur counterparts containing nominally the same content of glass formers (SiO_2_ and P_2_O_5_).

This decrease in density after introducing sulfur indicates a reduction in the rigidity of the glass matrix; the structure was weakened, which is favorable for higher cation mobility. According to [39], this will cause such materials to have a higher electrical conductivity.

The lower density of the sulfur-containing glass may be associated with the S speciation in the glass, resulting in a rather large range of possible (effective) ionic radii, ranging from 1.84 Å for the reduced sulfide [40] to 2.50 Å for the oxidized sulfate (SO_4_^2−^) [41]. The presence of the large anions S^2−^, SO_4_^2−^, and SO_3_^2−^ in their structures results in a loosening of their initial structures. The dark brown and red color of the sulfur-doped glass confirms the presence of reduced forms of sulfur in the form of S^2−^ in their structure, but the presence of other forms of sulfur cannot be excluded. According to the literature [37], sulfur–oxygen compounds are colorless, and the chromophores of the glass color may be elemental sulfur atoms or forms of sulfide.

The increase in the density of the 36Si11P sample may be because the rearrangement of ions caused structural changes that resulted in the formation of glass ceramics, i.e., the ions were more closely packed than in glass [42].

The molar volumes of the prepared glass, which were calculated based on the experimental compositions found with XRF, are shown in Table 2.

The molar volumes of the sulfur-free 45Si11P, 43Si4P, and 39Si8P glass samples were 23.8145, 24.9547, and 27.4620 cm^3^/mol, respectively. On the other hand, the sulfur-doped glass samples were characterized by lower molar volume values compared to their sulfur-free counterparts, which contained nominally the same content of glass formers; these values were within the range of 25.9264–23.5923 cm^3^/mol.

The decrease in the molar volume of the sulfur-doped samples in relation to the molar volume of the sulfur-free glass samples may be due to the formation of bridging oxygen (BO) and the compression of the network structure of the sulfur-doped glass samples.

In most glass samples, it was observed that the density and molar volume showed behaviors that were quite opposite to each other [43,44]. In contrast, a decrease in the density value, which was also accompanied by a decrease in the value of the molar volume, was noted by Kieldsen et al. [45] in a series of MgO/CaO sodium aluminosilicate glass by substituting MgO for CaO, as well as by Samdani et al. [46], who studied the mixed alkaline effect in double-alkaline borate glass from the MgO-BaO-B_2_O_3_-CuO system.

#### 2.2.2. Optical Basicity

The optical basicity is used to measure the ability of glass to donate the negative charge of an ion (the electron density carried by oxygen) [47].

The theoretical optical basicity (Λ_th_) of the studied glass samples was calculated by using the following relation:

Λ_th_ = X_SiO2_Λ_SiO2_ + X_P2O5_Λ_P2O5_ + X_K2O_Λ_K2O_ + X_MgO_Λ_MgO_ + X_SO3_Λ_SO3_ (1)

where X_SiO2_, X_P2O5_, X_K2O_, X_MgO_, and X_SO3_ are equivalent fractions of SiO_2_, P_2_O_5_, K_2_O, MgO, and SO_3_ based on the amount of oxygen and Λ_SiO2_, Λ_P2O5_, Λ_K2O_, Λ_MgO_, and Λ_SO3_ are optical basicity values assigned to the individual oxides, respectively. The values of Λ_SiO2_ = 0.48, Λ_P2O5_ = 0.33, Λ_K2O_ = 1.4, Λ_MgO_ = 0.78, and Λ_SO3_ = 0.25 were obtained from the literature [48].

The values of theoretical optical basicity (Λ_th_), which were calculated based on glass composition determined with XRF, are listed in Table 2.

The theoretical optical basicity Λ_th_ for the sulfur-free 45Si2P, 43Si4P, and 39Si8P glass samples was 0.6510, 0.6314, and 0.6096, respectively. On the other hand, the value of Λ_th_ for the sulfur-doped glass samples was in the range of 0.6229–0.5859. Therefore, the addition of sulfur had an effect on reducing the theoretical optical basicity.

According to the literature, the decrease in the theoretical optical basicity is caused by the decrease in the covalency of the cation–oxygen bonds of the studied glass [49], but this value may be useful in predicting the “trends” in optical basicity rather than the “true” optical basicity values.

### 2.3. Structural Studies

#### 2.3.1. Infrared Spectroscopy Results

**Figure 4:**
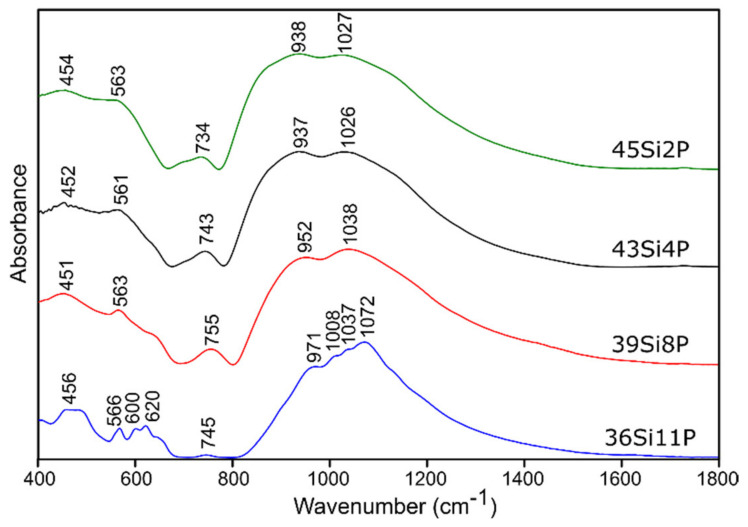
MIR spectra of the pure silicate–phosphate glass samples.

**Figure 5:**
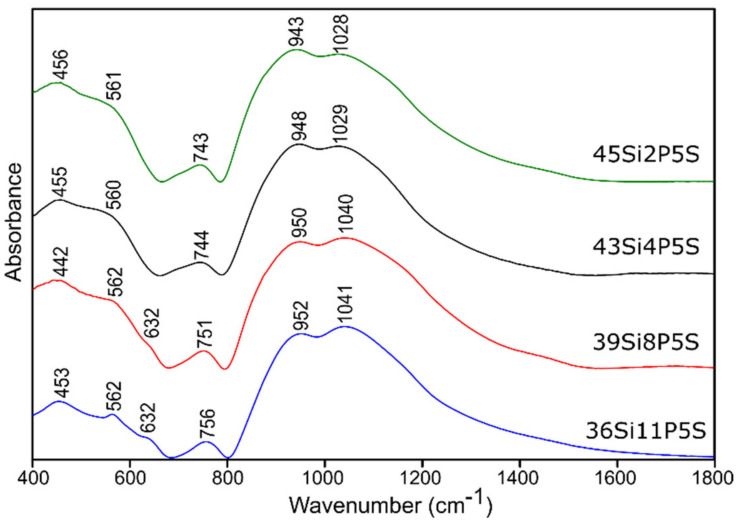
MIR spectra of the sulfur-doped silicate–phosphate glass samples.

The MIR absorption spectra in the range of 400–1800 cm^−1^ of all of the investigated glass samples (both pure and doped with sulfur) are shown in Figure 4 and Figure 5, respectively.

In general, the MIR spectra of all of the investigated glass samples exhibited three broad absorption bands in the region of 400–1800 cm^−1^, except for one composition, 36Si11P, which exhibited crystallization immediately after the cooling of the glass melt.

It is well known [50,51,52] that the absorption bands originating from silico-oxygen and phospho-oxygen subnetworks overlap in MIR spectra in both high- and low-frequency regions in the 400–1800 cm^−1^ range. The significant broadening of the bands in the MIR spectra of the studied glass samples that was observed made it impossible to identify the presence of the bands derived from sulfur ions. In the higher frequency range of 1080–1130 cm^−1^ (typically), as well as at 610–680 cm^−1^, sulfate ions may appear [53]. The problem is that, especially in the typical range, which should first have an intense absorption band and then a broad absorption band from the sulfate ions, there are absorption bands from silicate ions (900–1100 cm^−1^) and phosphate ions (1000–1100 cm^−1^) [50,51,52]. Absorption bands derived from the disulfides may occur in the range of 600–620 cm^−1^, but those from the polysulfides occur in the range of 470–500 cm^−1^ [53,54].

**Figure 6:**
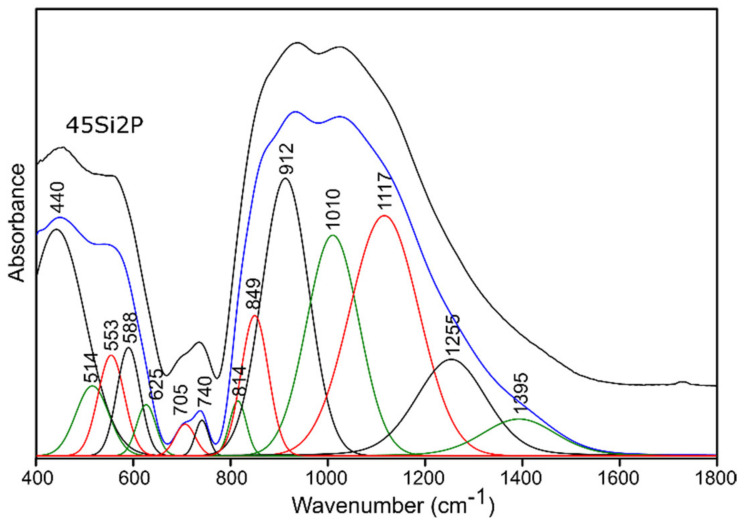
Deconvolution of the MIR spectra of the 45Si2P glass sample.

**Figure 7:**
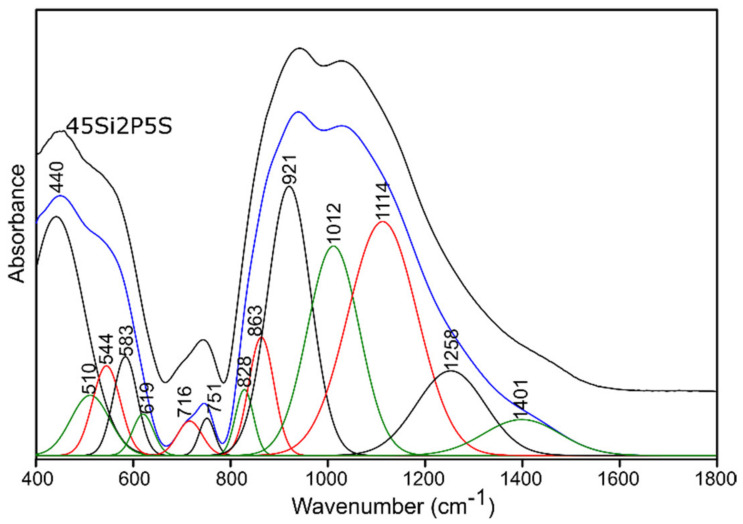
Deconvolution of the MIR spectra of the 45Si2P5S glass sample.

**Figure 8:**
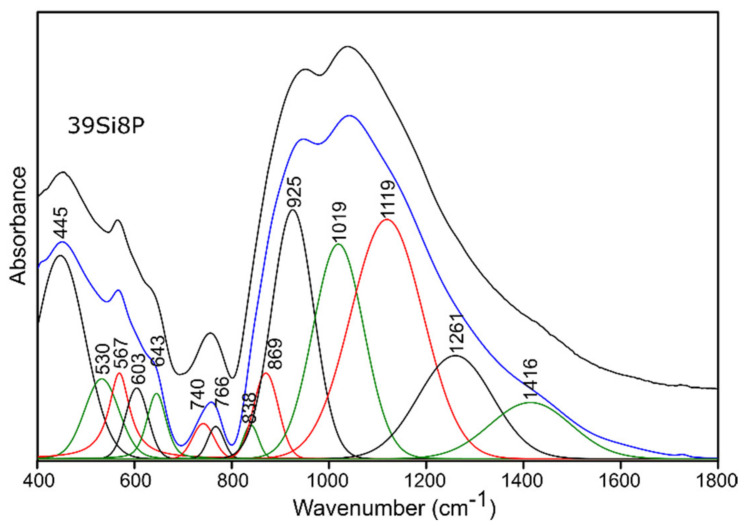
Deconvolution of the MIR spectra of the 39Si8P glass sample.

**Figure 9:**
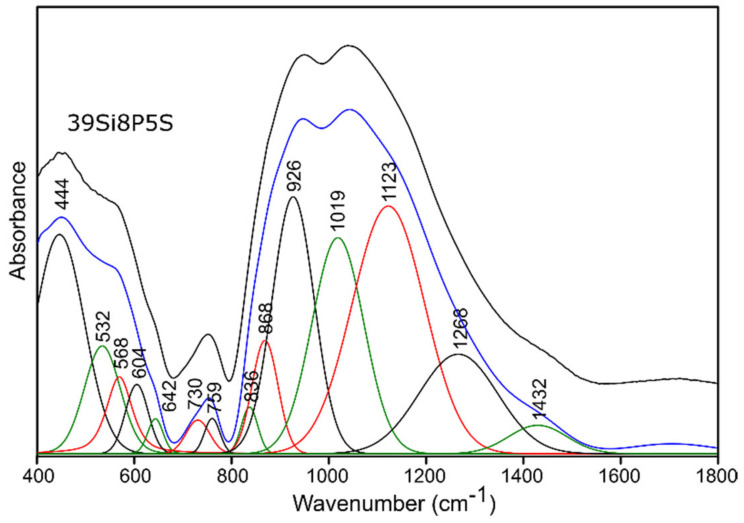
Deconvolution of the MIR spectra of the 39Si8P5S glass sample.

For this reason, and because of the significant asymmetry of the bands in MIR spectra of all of the studied glass (Figure 4 and Figure 5), the deconvolution of the MIR spectra was conducted in the range of 400–1800 cm^−1^ for the selected glass samples, and the results are presented in Figure 6, Figure 7, Figure 8 and Figure 9, while the exact assignments of the bands are presented in Table 3.

##### Pure Glass

The deconvolution of the MIR spectra of the pure 45Si2P glass sample in the 400–650 cm^−1^ region showed five Gaussian bands centered at 440, 514, 553, 588, and 625 cm^−1^ (Figure 6). The IR absorption in the 400–650 cm^−1^ range can be assigned to Si-O-Si, O-Si-O, and O-P-O bending vibrations [50,51,52,55,56,57,58]. The bands with higher frequencies in the range of 650–760 cm^−1^ can be assigned to symmetric Si-O-Si and Si-O-P stretching vibrations [50,51,52], as well as to the symmetric stretching of Si-O with three or four bridging oxygens (Si-O 3-4BO) (Table 3) [56].

In the next range of wavenumbers, the 800–1600 cm^−1^ region showed seven Gaussian bands centered around 814, 849, 912, 1010, 1117, 1255, and 1395 cm^−1^ (Figure 6). The band around 814 cm^−1^ was identified as the Si-O-Si bending vibration, in which the oxygens moved approximately at right angles to the Si-Si lines and in the Si-O-Si planes [58,59]. The bands at about 849 and 912 cm^−1^ can be assigned to the stretching modes of Si-O with two non-bridging oxygens per SiO_4_ tetrahedron (Si-O-2NBO) [56,60]. These bands are characteristic of silicate glass materials that are modified through the incorporation of alkali and/or alkali Earth elements.

According to the literature [55,56,60], the absorption band centered at 1010 cm^−1^ can be assigned to the stretching modes of Si-O with one non-bridging oxygen per SiO_4_ tetrahedron (Si-O-1NBO) [56,60], as well as to the asymmetric stretching vibrations of PO_4_^3-^ units [55].

The band centered at 1117 cm^−1^ can be assigned to Si-O-Si stretching [50] or to the symmetric stretching vibrations of O-P-O bonds [61].

The IR absorption at around 1255 cm^−1^ (Figure 6) can be assigned to the Si-O-Si stretching mode and is referred to as a characteristic of glass with higher SiO_2_ mol.% content [58,62]. This band may also be associated with the asymmetric stretching of O-P-O bonds [61], but this is less likely. The origin of the band at about 1395 cm^−1^ in the spectrum of the 45Si2P glass is difficult to identify, but considering data from the literature [57,63], it may come from the vibrations of P=O bonds.

In the case of the studied glass samples, introducing an increasing amount of P_2_O_5_ (up to 8 mol.%) did not reveal the presence of any additional absorption bands among the pure glass materials. This behavior was also observed in [64]. The observed change, which resulted from the increasing addition of P_2_O_5_ to the composition of the studied glass samples, concerned the reduction in the half-width of the absorption bands at higher wavenumbers (800–1600 cm^−1^) (Figure 4). The increase in the addition of P_2_O_5_ of up to 8 mol.% (pure 39Si8P glass sample) also caused a decrease in the band intensity at about 912 cm^−1^ for the 45Si2P glass sample. Moreover, this band shifted towards higher wavenumbers (912 → 925 cm^−1^) in the case of the 39Si8P glass sample (Figure 8).

In the range of the mean values of the wavenumbers, the addition of 8 mol.% P_2_O_5_ caused an increase in the intensity of the bands at about 514, 625, and 705 cm^−1^ (Figure 6 and Figure 8), while the addition of P_2_O_5_ caused the band intensity to decrease to about 440, 553, and 588 cm^−1^ for the 45Si2P glass sample. The most noticeable, however, was the shift of all of the mentioned bands towards higher wavenumber values as a result of the increase in P_2_O_5_.

The shifts of the bands towards higher wavenumbers may be a result of the stronger bond strength and reduced mass of the connections with oxygen, as well as the replacement of ions with a lower electronegativity with ions with a higher electronegativity. The field strengths of the O^2−^ ions at a distance for Si^4+^, P^5+^, Mg^2+^, and K^+^ ions, which were determined as the ratio of Z/a^2^ (Z is the valence of the cation, and a is the distance between the cation and oxygen), were 1.57, 2.1, 0.45, and 0.13, respectively [65]. According to Görlich [65], the electronegativities of Si^4+^, P^5+^, Mg^2+^, and K^+^ are 1.82, 2.19, 1.05, and 0.56, respectively. The shifts towards higher wavenumbers in the pure glass samples were caused by the successive replacement of Si-O-Si bonds with Si-O-P linkages as the P_2_O_5_ content increased.

The spectra of the glassy crystalline material 36Si11P, which contained the highest P_2_O_5_ content (nominally 11 mol.%), were not subjected to the decomposition process (Figure 4). The spectra of this glass showed a series of bands related to the crystallization of KMgPO_4_ in its structure, such as the bands at about 460, 620, 971, 1008, and 1072 cm^−1^ [55].

##### Sulfur-Doped Glass

The decomposition process of the MIR spectra of the sulfur-doped glass (Figure 5) did not reveal the presence of additional bands resulting from connections with sulfur (Figure 7 and Figure 9, Table 3). However, the nominal introduction of 5 mol.% of SO_3_ into the pure 45Si2P glass structure caused a shift of the band at about 912 cm^−1^ towards a higher wavenumber of 921 cm^−1^. This behavior was also noted for the bands at about 814, 740, and 705 cm^−1^ (to 828, 751, and 716 cm^−1^, respectively), which may indicate an increase in the degree of polymerization of the silicate–phosphate network.

In the MIR spectra after decomposition, the addition of SO_3_ in the nominal amount of 5 mol.% to the structure of the 39Si8P glass at the expense of MgO, as mentioned above, did not affect the appearance of additional bands that resulted from connections with sulfur (Figure 9). However, the addition of sulfur influenced the changes in the position and intensity of the bands in the range of lower frequencies. Particularly noteworthy is the increase in the intensity of the band at about 445 cm^−1^ and the weaker band at about 530 cm^−1^ because, indirectly, this may also suggest an increase in the degree of polymerization of the analyzed glass structure.

As is seen in the XRD analysis, the addition of SO_3_ in the nominal amount of 5 mol.% at the expense of MgO caused an increase in the formation abilities of glass with the highest content of P_2_O_5_ (the 36Si11P5S material) (Figure 1). The addition of SO_3_ inhibited the crystallization process immediately after the cooling of the glass melt. We did not observe any sharp, high-intensity bands in the MIR spectra of this glass, which would be typical for the crystalline phase of KMgPO_4_ (Figure 5). Perhaps the addition of SO_3_ caused a greater distribution of phospho-oxygen tetraeders in the structure of these glass, thus preventing the crystallization of KMgPO_4_. Moreover, based on [66], the appearance of sulfur species requires K^+^ ions in order to balance the charge; they are sourced from the silicate–phosphate network.

In [66]—in which the subjects of the research were glass samples with nominal chemical compositions of 41SiO_2_-6P_2_O_5_-20K_2_O-28MgO-5SO_3_ in mol.% that were obtained under different reducing conditions with Raman spectroscopy coupled with deconvolution of the resulting spectra—it was shown that the introduction of sulfur significantly altered the Raman spectra in the range of 250–540 cm^−1^. The appearance of several bands in this area was attributed to the presence of reduced sulfur species, such as the polysulfide K_2_S_n_, which was probably in the form of K_2_S_6_, in the structure. The absence of bands derived from sulfur ions in the MIR spectra and their appearance in the Raman spectra may be related to the low polarizability of bonds with sulfur [67]. However, the addition of sulfur at the cost of magnesium has an influence on the positions of the bands in the MIR spectra and mainly causes them to shift towards higher wavenumbers. This indicates that the introduction of sulfur causes polymerization of the silicate–phosphate network through the accumulation of potassium near the sulfur, as stated earlier in [66]. There are reports in the literature [8] that the next-nearest neighbor cations of SO_4_^2−^ groups will most likely be modifier cations, and the same applies to reduced sulfur ions (S^2−^), which may additionally confirm our assumptions.

In the case of these types of sulfur-doped glass, Raman spectroscopy can be useful in determining the valency state of sulfur in their structures, but the best method of determining the valency state of sulfur and its local structural environment is the X-ray absorption near edge structure XAFS method, the results of which will be the subject of research in the near future.

### 2.4. Optical Studies

**Figure 10:**
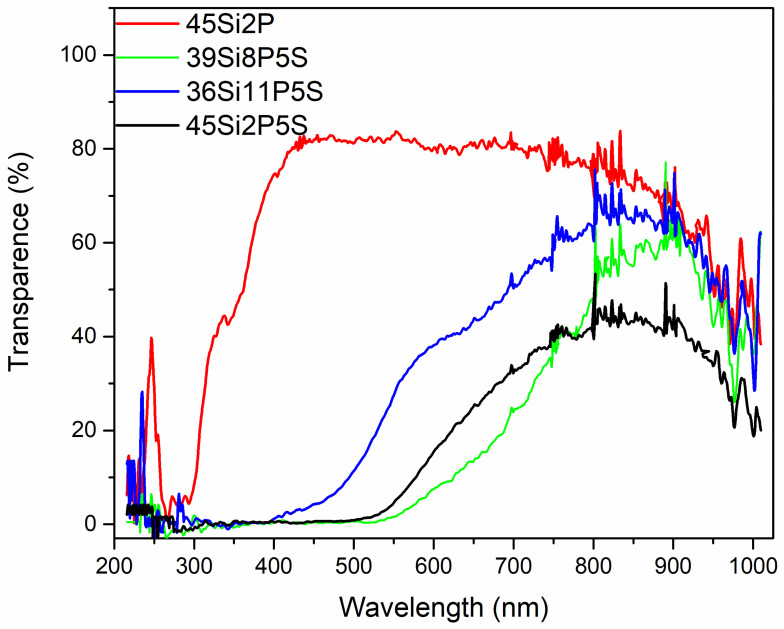
Transparency spectra of the silicate–phosphate glass samples: 36Si11P5S (0.5 mm), 39Si8P5S (0.5 mm), 45Si2P5S (0.5 mm), and 45Si2P (2 mm).

**Figure 11:**
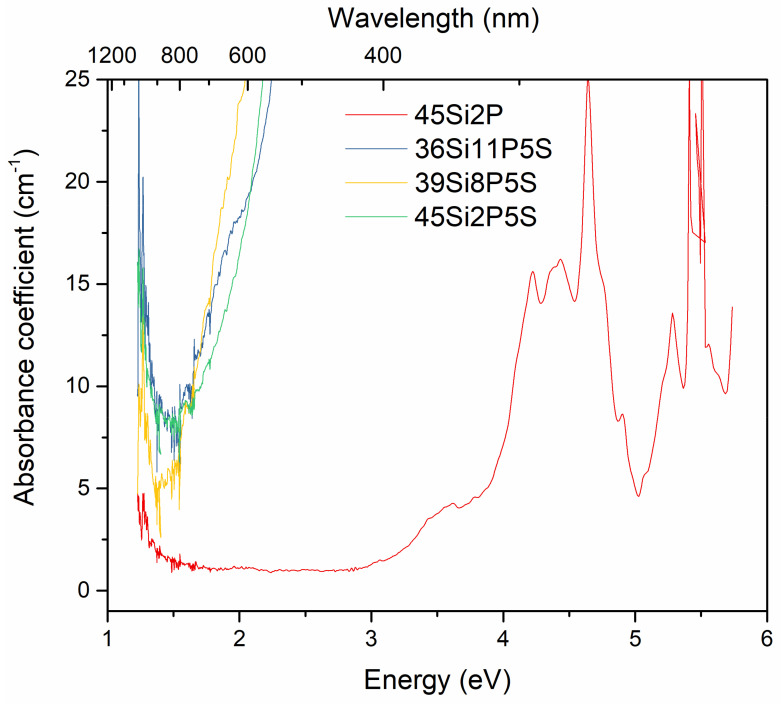
Absorbance coefficient spectra of the silicate–phosphate glass samples: 36Si11P5S, 39Si8P5S, 45Si2P5S, and 45Si2P.

The optical transmittance and absorbance spectra of the investigated 36Si11P5S, 39Si8P5S, 45Si2P5S, and 45Si2P glass samples were measured at room temperature. Figure 10 and Figure 11 depict the transmittance (as obtained) and absorbance coefficient spectra of the selected sulfur-containing glass samples (36Si11P5S, 39Si8P5S, and 45Si2P5S) compared to the transmittance and absorbance spectra of the undoped glass sample (45Si2P). The absorption coefficient α(ν) depends on the material and on the wavelength of light that is passing through the material. This parameter can be calculated with Equation (2):

(2) $$\alpha =\frac{1}{d}ln\frac{I_{0}}{I_{T}}=\frac{1}{d}ln(\frac{1}{T})$$

where *d* is the optical path of the sample, *T* is the transparency, and *I_o_* and *I_T_* are the intensities of the incident and transmitted radiation, respectively.

The absorbance coefficient is necessary for calculating the extinction coefficient according to the following equation:

(3) $$k=\frac{\alpha \lambda }{4\pi }$$

where *k* is the extinction coefficient, which is the imaginary part of the complex refractive index of the material, α is the absorption coefficient, and *λ* is a variable wavelength.

To elucidate the dependence of the optical properties of the selected glass, the transparency T(%) and absorbance coefficient spectra *α* (cm^−1^) in a wide spectral region covering the range from 200 to 1100 nm were measured for all of the synthesized glass.

Figure 10 shows the spectral dependence of the transparency of the 36Si11P5S, 39Si8P5S, and 45Si2P5S glass samples on different concentrations of sulfur. In our glass samples, sulfur was trapped through physically occlusion during the melting processes of the glass and was retained in the form of sulfide ions because reducers were added to the multi-component raw material mixes. Nevertheless, the presence of the sulphate ions that are usually formed under oxidizing conditions cannot be excluded. Thus, the transparency and color of these sulfur-containing glass samples were mainly determined by the sulfide ions. The absorption coefficients plotted as a function of the wavelength and energy are shown in Figure 11. Figure 11 indicates that the investigated glass samples had relatively low transmittance in this region. The glass samples were colorless, and had the optical transparency of commercial fused silica glass. The decrease in transparency in the IR spectrum should be associated with the presence of elements such as sulfur and structural defects. The sulfur-doped glass samples exhibited an absorption edge in the low-energy region (E = 2.5 eV or λ = 600 nm) (Figure 10 and Figure 11). However, these glass samples exhibited a relatively high transparency, ranging from 40% to 60% in the infrared region (E = 3.5 eV, λ > 750 nm). Interestingly, the absorption region generally shifted to the IR region as the sulfur content in the glass samples constantly increased (Table 3). This shift can be directly attributed to the sulfur content in the glass samples, the structural disorder, and the numbers of vacancies and clusters, i.e., small agglomerations of atoms and molecules. As can also be seen from Figure 11, with increasing sulfur content in the glass samples, the determined absorption edge became more vertical. No absorption bands were observed in these spectra. Such significant variations in a glass’s features cause a considerable increase in the optical band-gap energy.

**Figure 12:**
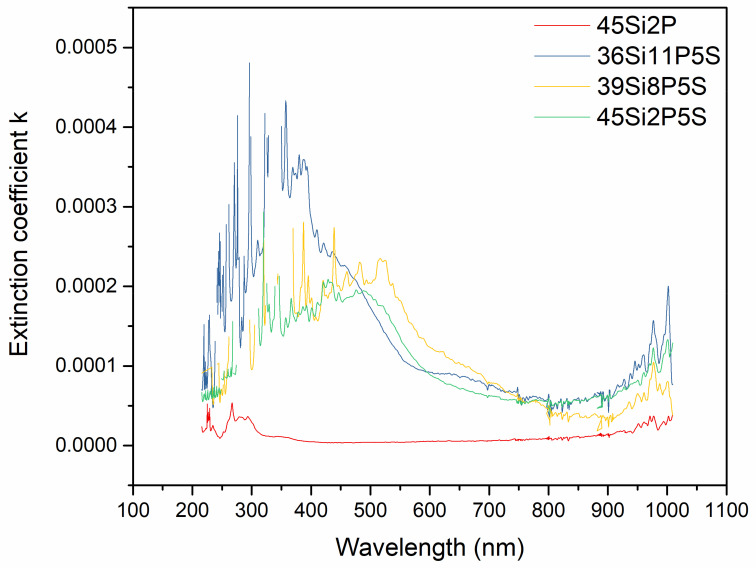
Extinction coefficient vs. wavelength for the silicate–phosphate glass samples: 36Si11P5S, 39Si8P5S, 45Si2P5S, and 45Si2P.

Figure 12 shows the *k*-parameter as a function of the wavelength of the incident light of the investigated glass. Our results indicate that the *k*-value of the undoped material decreased drastically and abruptly as it approached zero in a wide spectral range (400–700 nm).

As the doping concentration of sulfur and other ions incorporated into the glass increased, the *k*-value of the 45Si2P glass sample decreased slightly; the doped glass displayed energy loss due to absorption and/or scattering. The change in *k*-value for 45Si2P confirmed its good transparency.

**Figure 13:**
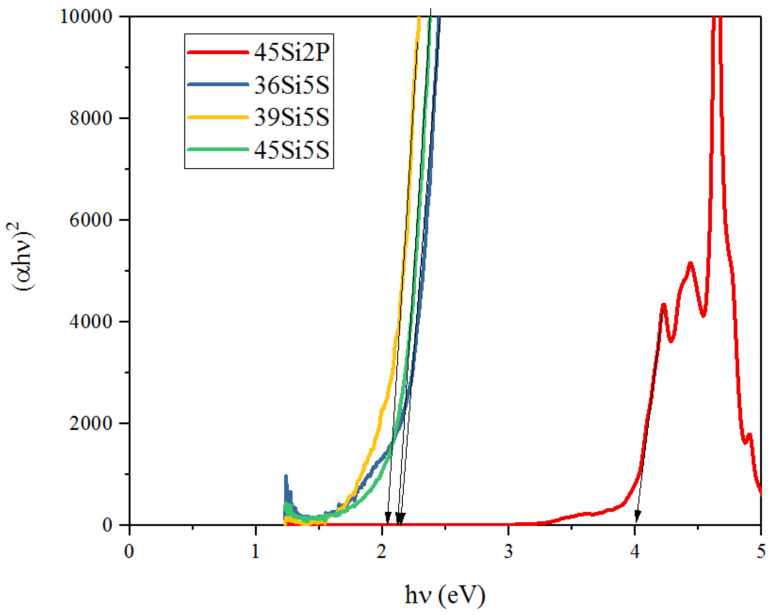
Tauc plot for the silicate–phosphate glass samples: 36Si11P5S, 39Si8P5S, 45Si2P5S, and 45Si2P; *n* = 2.

The band-gap energy, *E_g_*, was determined by using Tauc’s well-known equation [68]:

(4) $$\alpha h\nu =B{\left(h\nu -E_{g}\right)}^{n},$$

where *α* is the absorption coefficient (cm^−1^), *h* is the Planck constant (eV⋅s), *ν* is the frequency of light (Hz), *C* is an energy-independent value, and *n* has different values—that is, 1/2, 2, 1/3, and 3, corresponding to directly allowed, indirectly allowed, directly forbidden, and indirectly forbidden transitions, respectively—in amorphous materials. The band-gap energy can be estimated by plotting αhν against hν and extrapolating the fitting line to its intercept with the hν axis. For the glass samples under consideration, the fitted Tauc plot is shown in Figure 13.

Thus, the glass system presented here showed indirectly allowed transitions, and the values are listed in Table 4. The values of the band-gap energy were found to decrease with increases in sulfur content. This may be attributed to the increase in bridging oxygen in this glass system. The values presented in Table 3 are also in good agreement with the trend followed by the band-gap energy.

**Figure 14:**
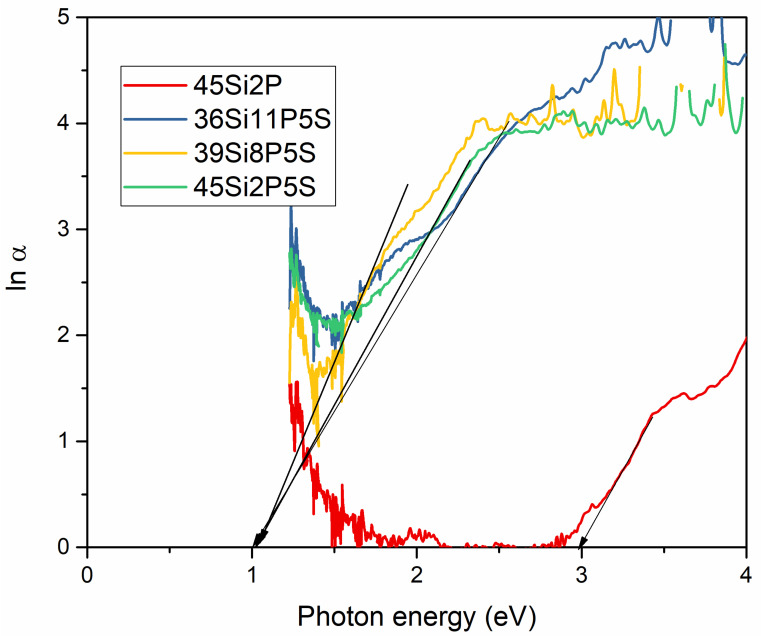
Urbach plot for the silicate–phosphate glass samples: 36Si11P5S, 39Si8P5S, 45Si2P5S, and 45Si2P.

In our work, we also calculated the Urbach energy (*E_U_*), and the results are presented in Figure 14. It is commonly known that the Urbach energy provides information about concentrations of defects in materials. Materials with large Urbach energies exhibit a greater tendency to convert weak bonds into defects. The Urbach energy also corresponds with the widths of localized states, and it can be used to characterize the degree of disorder in amorphous materials. The Urbach energies of the samples were estimated according to Equation (5), and least-square fitting of *Lnα* against the *hν* curves in the tailing parts of the localized states was performed:

(5) $$\alpha =\beta exp\left(\frac{h\nu }{E_{u}}\right)$$

which can be rewritten as follows:

(6) $$ln\alpha =\frac{h\nu }{E_{u}}+ln\beta$$

which represents the Urbach energy values of the fused glass with different concentrations of sulfur. The Urbach energies were estimated to be ca. 2.98, 1.05, 1.05, and 1.04 eV for glass samples 45Si2P, 36Si11P5S, 39Si8P5S, and 45Si2P5S, respectively. As can be concluded, the incorporation of sulfur reduces the Urbach energy value.

### 2.5. Photoluminescence Properties

**Figure 15:**
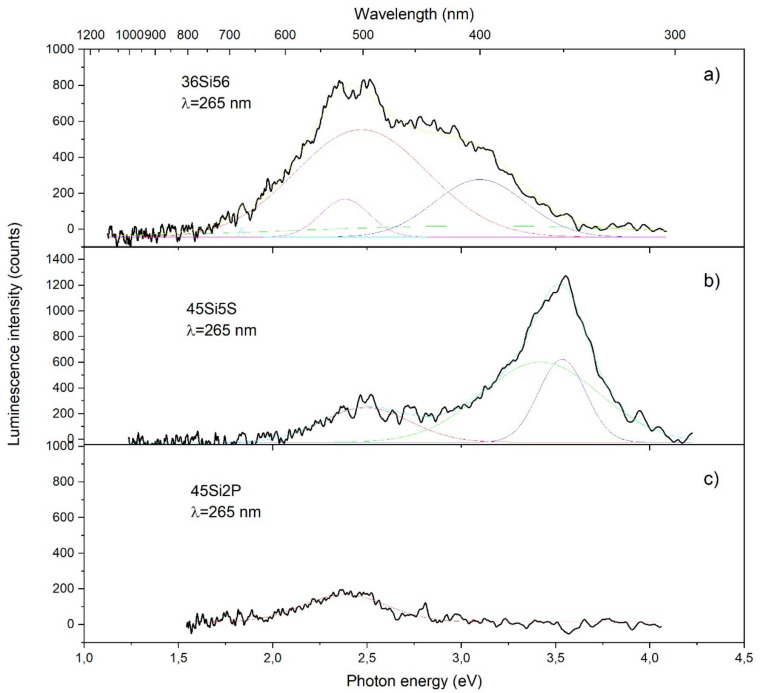
Luminescence spectra for the 36Si11P5S (**a**), 45Si2P5S (**b**) and45Si2P (**c**) glass samples with deconvolution.

The photoluminescence (PL) emission properties of the prepared glass samples were studied in three spectral regions: ultraviolet, visible, and NIR. Due to the complexity of the chemical elements and microscopic details, it seemed difficult to determine the exact mechanisms that were responsible for these visible emissions. Moreover, the spectroscopy of the color centers in the glass samples was difficult because of the inhomogeneous broadening and absence of both translational and orientational orders. The emission spectra of the prepared samples are shown in Figure 15. In this paper, we report an investigation of the photoluminescence spectra of four different types of glass materials: 45Si2P, 36Si11P5S, 39Si8P5S, and 45Si2P5S. The results of this study show that each of the samples had a different characteristic luminescence spectrum. In the case of the 36Si11P5S glass sample, luminescence was not detected in the observed region.

Figure 15 shows the PL spectra of the three aforementioned samples after excitation with a 265 nm laser beam. After the measurement, the spectra of the samples were deconvoluted with a Gaussian function. In spectrum (Figure 15a), three strong and overlapping emission bands were observed, with maximums at 450, 490, and 530 nm. The small peaks located at 530 nm could be the second harmonic of the laser pump. In the case of Figure 15b, it can be observed that there was one strong luminescence peak at 345 nm and a second at 510 nm. In the last spectrum (Figure 15c), a relatively small peak appeared at 520 nm. Spectral analyses suggested that the green and yellow emissions arose from optical transitions in a single ionized oxygen vacancy (Vo 1) and the single negatively charged interstitial oxygen ion (Oi 2) inside the matrix [69]. According to Skuja et al. [70], in silica glass, absorption between 210 and 240 nm corresponds to the E’ color center. Furthermore, in the case of glassy silica, both vacancy-type E’ centers and dangling bonds are expected to co-exist. The presently accepted model for the E’ center in α-quartz features an asymmetric relaxation of the oxygen vacancy. In addition, peroxy radicals can be taken under consideration as color centers.

The UV emissions with a maximum at 340 nm could be attributed to the free-excitonic transition or defects in the silica, feldspars, or feldspathoids. The strained Si–O structures include some nonbridging oxygen or silicon vacancy–hole centers and Si–O bonding defects, which seem to be responsible for the 340 nm emissions [71]. The spectra recorded for the 36Si11P5S glass sample under excitation with photons of the highest energy showed a wide luminescence from 300 to 600 nm.

The experimental results show that the intensities of the green and yellow PL bands obtained were strongly affected by the width of the free-carrier depletion region at the particle surface [72].

**Figure 16:**
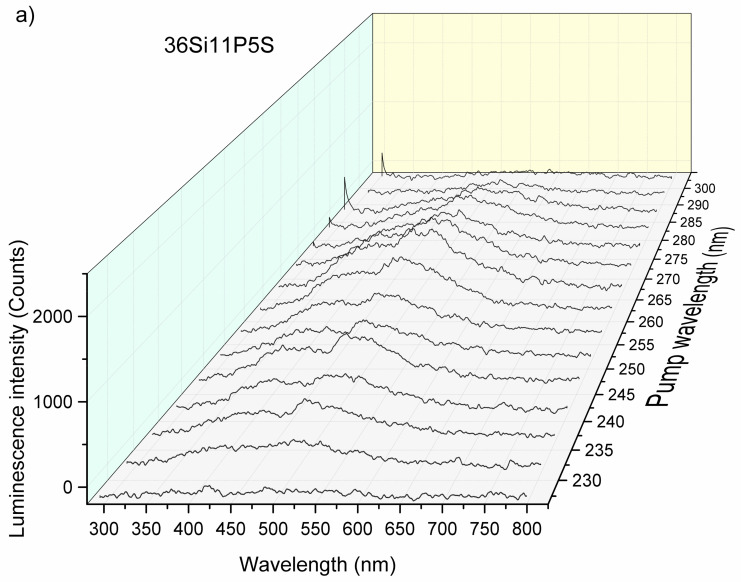

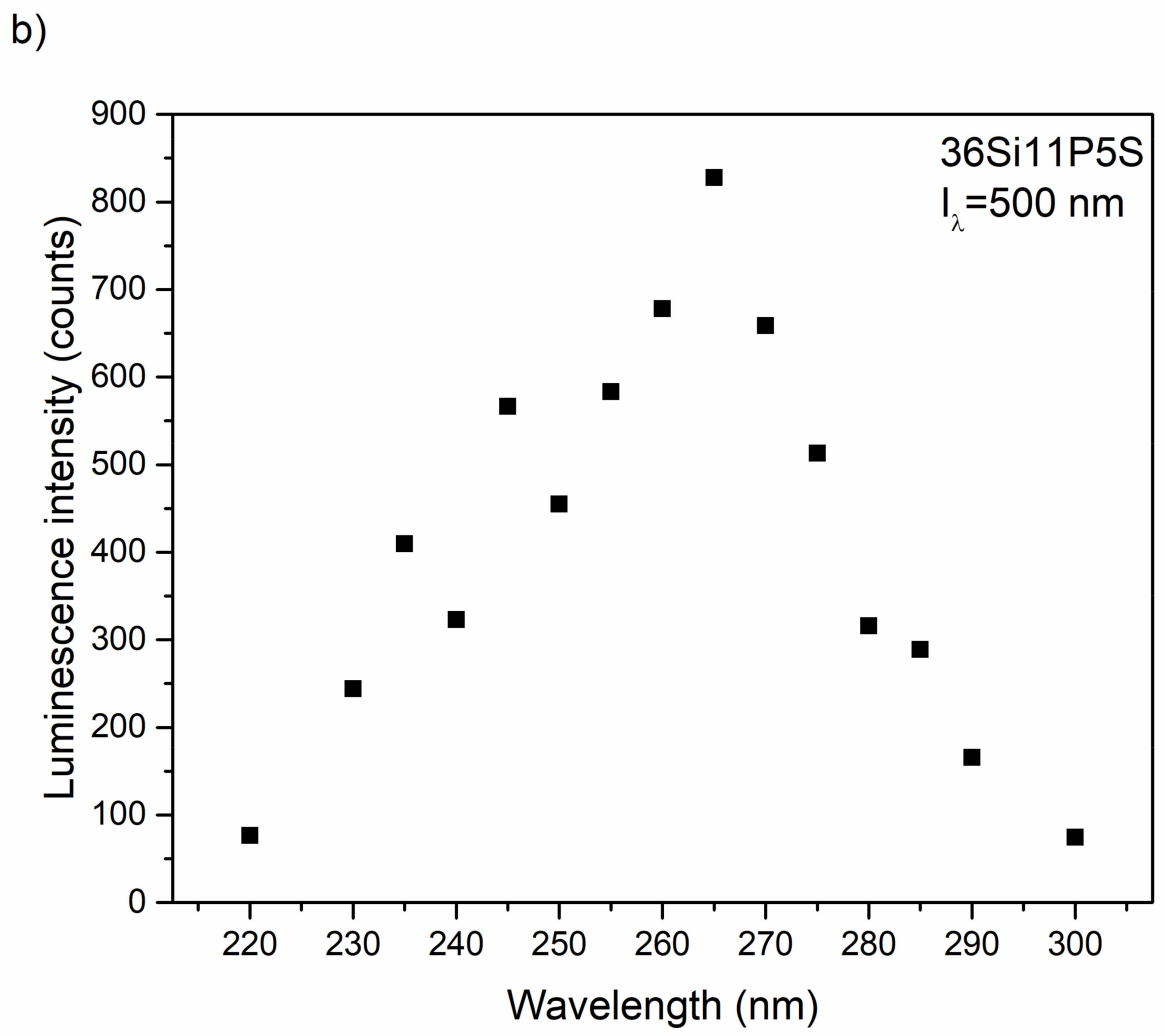
Luminescence intensity vs. wavelength and the laser pump (**a**) and luminescence intensity vs. wavelength at 500 nm (**b**) for the 36Si11P5S sample.

**Figure 17:**
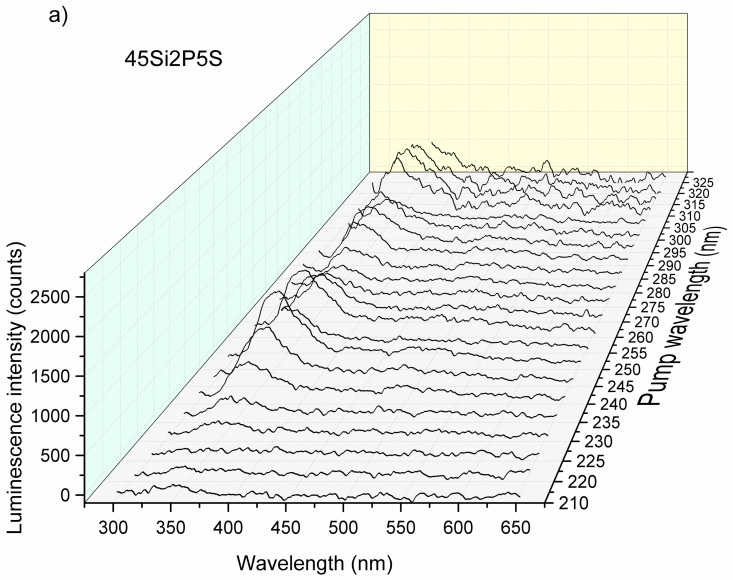

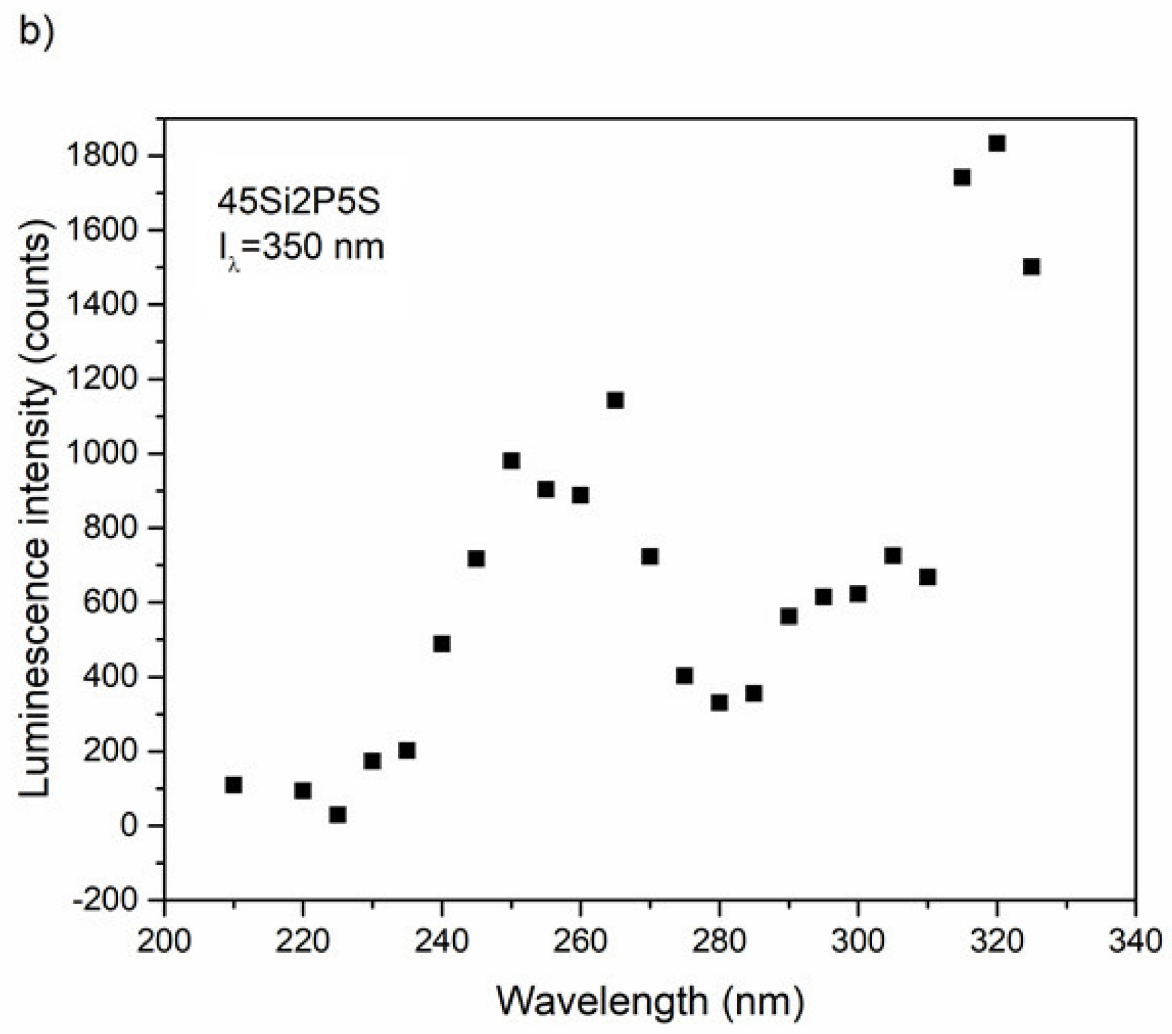
Luminescence intensity vs. wavelength and the laser pump (**a**) and luminescence intensity vs. wavelength at 500 nm (**b**) for the 45Si2P5S sample.

**Figure 18:**
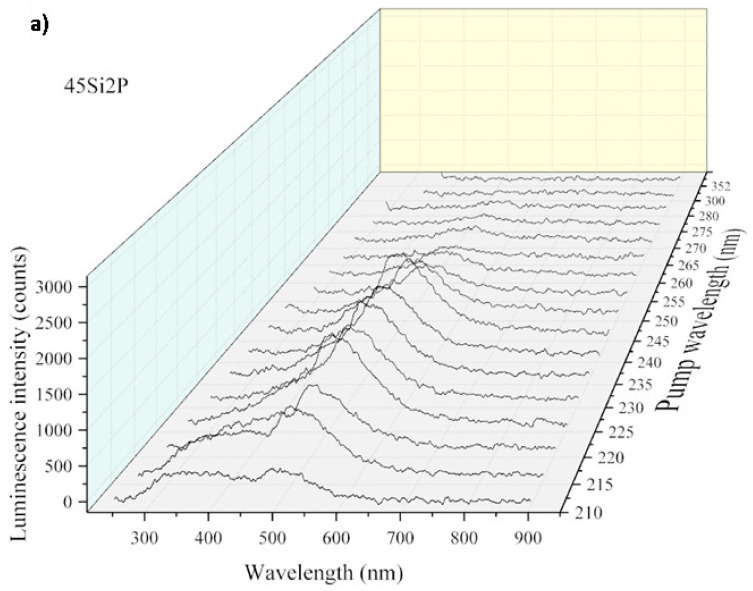

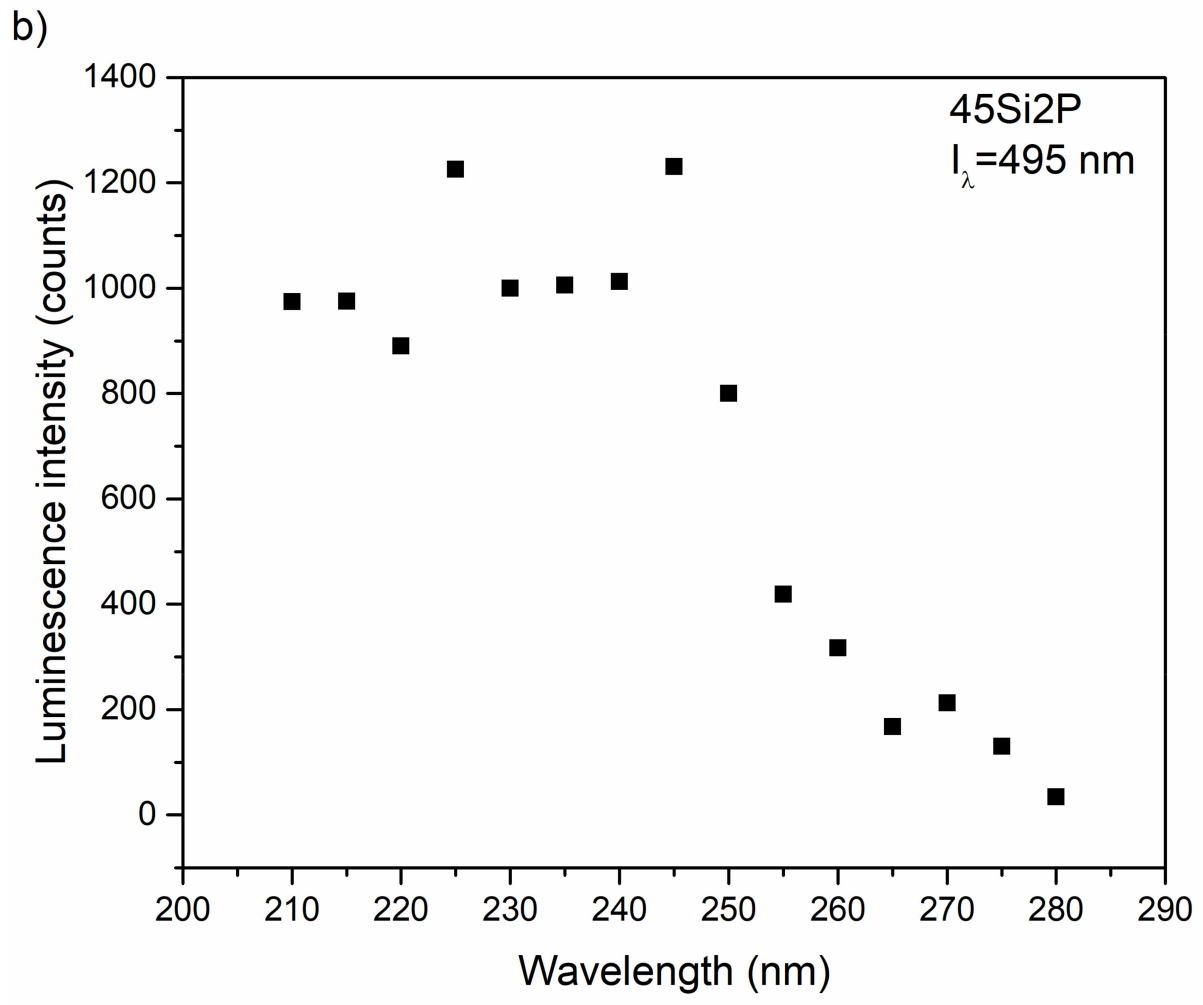
Luminescence intensity vs. wavelength and the laser pump (**a**) and luminescence intensity vs. wavelength at 500 nm (**b**) for the 45Si2P sample.

The intensity of the luminescence spectra of the glass samples also depended on the excitation source. To take advantage of this fact, we performed measurements by sweeping the wavelength of the laser pump from 210 to 300 nm with steps of 5 nm. The evolution of the luminescence efficiency with respect to the wavelength as a function of the pump is presented in Figure 16a, Figure 17a, and Figure 18a.

Figure 16, Figure 17 and Figure 18 show an overview of the diffuse excitation spectra of the sulfur-containing 36Si11P5S, 39Si8P5S, and 45Si2P5S glass samples when the wavelength of the laser excitation was changed from 210 to 400 nm. All of the spectra are discussed in comparison with undoped samples. Through a simple comparison of these results, it can be shown that the spectral shapes of the investigated glass samples were influenced by the different excitation mechanisms.

The luminescence intensity of the 36Si11P5S sample under a 265 nm laser beam was very strong. For better readability, Figure 16b, Figure 17b, and Figure 18b show the dependence of the luminescence intensity on the selected wavelength as a function of the excitation beam’s wavelength. However, it should be considered that the strong signal recorded for excitation with a laser beam with a length of more than 300 nm may be overestimated by the spectrum of the laser beam.

In the case of the 45Si2P5S sample, two spectral regions where the luminescence of the glass was the highest can be observed, as shown in Figure 17. The first maximum of the stimulated emission intensity was located in the UV region at 250 nm and the second one is located at ca. 310 nm. As the photon energy decreased, the peak luminescence also decreased to 300 nm. According to Kasymdzhanov, the luminescence observed in the wide spectral range from UV to NIR in quartz glass could be due to the presence of M centers that were created during the melting process [73].

The determination of the value of the spectral maximum at about 600 nm was a difficult task because of the effect of Raman scattering. In summary, the number of transition levels that contributed to the stimulated emissions decreased as the photon energy of the irradiation decreased.

The measurement error for this experiment was 10% because the influence of the optical system on changes in the laser beam’s power could not be precisely determined for wavelengths below 250 nm. Additionally, the power of the laser beam achieved on the surface of the samples was not constant in every repetition. When exposed to the laser beam, the samples were heated, so the photoluminescence could have been changed by the increased temperature. More detailed experiments should be performed in a vacuum system. Nevertheless, the obtained results indicate the necessity of conducting further research that is focused on the full characterization of the spectra obtained here, including an unequivocal explanation of the luminescence mechanism. Further investigations, including time-resolved spectroscopy and EPR studies of temperature-dependence luminescence, are very necessary and will be shown in future research.

## 3. Materials and Methods

### 3.1. Glass Synthesis and Compositional Analysis

A series of pure and sulfur-doped silicate–phosphate glass samples with nominal compositions of (47-x)SiO_2_·xP_2_O_5_·20K_2_O·33MgO and (47-x)SiO_2_·xP_2_O_5_·20K2O·28MgO·5SO_3_ (x = 2, 4, 8, 11 mol.%) were prepared. The starting materials were analytical-grade (>99% purity) SiO_2_, (NH_4_)_2_HPO_4_, K_2_CO_3_, MgO, and K_2_SO_4_. All of the batches containing sulfate included activated carbon as a reducer in an amount equivalent to the added K_2_SO_4_. The raw material mixtures were melted in ceramic crucibles in electric furnaces at 1723 K in air. The obtained melts were poured onto a steel plate.

The amorphous nature of the samples was verified using X-ray diffraction (Empyrean X-ray Diffractometer, Malvern Panalytical Ltd, Malvern, UK, with a Cu lamp in the 2θ range of 5–90°).

The actual chemical compositions of the synthesized glass samples were determined through X-ray fluorescence spectrometry (XRF) with an Axios mAX WDXRF X-ray fluorescence spectrometer with Rh lamp of power 4 kW (Panalytical, Malvern, UK); the glass samples’ compositions were normalized to 100%.

The homogeneity of the glass was determined through elemental mapping at the microstructural level by scanning electron microscopy (SEM) with energy-dispersive X-ray spectrometry (EDS) using a Phenom XL microscope (Thermo Fisher Scientific, Waltham, MA, USA).

### 3.2. Structural Characterization of the Glass

#### 3.2.1. Density and Molar Volume

The true densities (*d_r_*) of the samples were measured in an AccuPyc II 1340 pycnometer (Micromeritics Instrument Corporation, Norcross, GA, USA) at room temperature with helium as the probe gas. Each measurement collected datapoints from 30 cycles. Before the analysis, the samples were degassed at about 373 K c.

The molar volumes (*V_mol._*) of the glass samples [72] were calculated with Equation (7):

(7) $$V_{mol.}=\left(\overset{}{\underset{MO}{\sum }}x_{MO}M_{MO}\right)d_{r}^{-1}$$

where:

*x_MO_*—mole fraction of the given glass component in the form of the oxide (*MO*);
*M_MO_*—molar mass of the given glass component in the form of the oxide (*MO*);
*d_r_*—experimentally determined true density of a given glass (g·cm^−1^).

#### 3.2.2. Infrared Spectroscopy

IR spectroscopic measurements of the obtained glass were obtained with a Vertex 70v spectrometer (Bruker, Billerica, MA, USA) using the standard KBr pellet method. A total of 128 scans with a resolution of 4 cm^−1^ were accumulated in the range of 400–4000 cm^−1^ (Middle Infrared). The positions of the bands were determined in accordance with the second derivative with the Bruker OPUS software. (version 7.2, FT-IR Spectroscopy Software, Bruker, Billerica, MA, USA). Spectral deconvolution was carried out by using the curve-fitting function in the OPUS software. The Levenberg–Marquardt algorithm was used to fit the component bands.

### 3.3. Optical and Luminescence Properties

The optical transmittance and absorbance spectra were determined at room temperature. Figure 10 and Figure 11 depict the transmittance (as obtained) and absorbance coefficient spectra of the selected glass (36Si11P5S, 39Si8P5S, and 45Si2P5S) compared to the transmittance and absorbance spectra of the undoped glass (45Si2P). The absorption coefficient *α*(*ν*) depends on the material and on the wavelength of the light passing through the material. This parameter can be calculated with Equation (8):

(8) $$\alpha =\frac{1}{d}ln\frac{I_{0}}{I_{T}}=\frac{1}{d}ln(\frac{1}{T}),$$

where *d* is the optical path of the sample, *T* is the transparency, and *I_o_* and *I_T_* are the intensities of the incident and transmitted radiation, respectively.

The absorbance coefficient is necessary for calculating the extinction coefficient according to the following equation:

(9) $$k=\frac{\alpha \lambda }{4\pi }$$

where *k* is the extinction coefficient, which is the imaginary part of the complex refractive index of the material, *α* is the absorption coefficient, and *λ* is a variable wavelength. A high-resolution (0.5 nm) spectrometer (Stellarnet SilverNova, Tampa, FL, USA) was employed to verify the optical properties of the glass, and a Xenon short-arc lamp (Instytut Fotonowy, 150 W, Kraków, Poland) was used as a light source.

The reflected luminescence spectra were obtained by using an optical parametric oscillator laser (EKSPLA PT304, Vilnius, Lithuania) after excitation between 210 and 300 nm at room temperature. The emission signals were also recorded with a SilverNova Stellarnet spectrometer (Stellarnet, Tampa, FL, USA).

## 4. Conclusions

A combined structural and optical study of a series of pure and sulfur-doped silicate–phosphate glass samples with varying SiO_2_/P_2_O_5_ content was reported in this paper. All of the compositions were able to form glass through the normal melt–quench process, except for the 36Si11P glass sample, which had a tendency toward crystallization after the cooling of its melt, which was possibly due to the lower Si/P ratio in its composition. The addition of sulfur increased the abilities of glass samples with higher amounts of P_2_O_5_ to form.

The sulfur-doped glass samples were characterized by a lower density value than that of their sulfur-free counterparts; therefore, their networks had less rigidity than the networks of their sulfur-free counterparts. The lower density of the sulfur-doped glass samples was probably related to the presence of large sulfur-containing anions in their structures—mainly S^2−^, but also SO_4_^2−^ and SO_3_^2−^ (the presence of other forms of sulfur was not excluded).

The addition of sulfur showed changes in the silicate–phosphate networks, making them more polymerized, which was probably related to the concentration of potassium ions in the vicinity of the sulfur ions.

Thus, it was concluded that luminescence was excited in the selected silicate–phosphate glass samples in both the UV and visible regions of the spectrum. The excitation efficiency of the wide luminescence in the investigated glass samples changed with the energy of the excitation wavelength, and a maximum point was obtained for one wavelength, which was different for each individual type of glass. The character of the luminescence changed not only for each type of silicate–phosphate glass, but also within series of the same types of glass, indicating the significance of the conditions of glass synthesis.

## Figures and Tables

**Table 1:** Experimental glass chemical compositions from XRF analysis (nominal value in brackets) in mol.%.

Comp.	45Si2P	45Si2P5S	43Si4P	43Si4P5S	39Si8P	39Si8P5S	36Si11P	36Si11P5S
SiO_2_	43.576 (45)	48.040 (45)	41.463 (43)	46.311 (43)	37.606 (39)	43.675 (39)	33.543 (36)	38.918 (36)
P_2_O_5_	2.313 (2)	1.238 (2)	4.624 (4)	0.796 (4)	9.041 (8)	3.802 (8)	12.380 (11)	7.034 (11)
K_2_O	19.407 (20)	16.385 (20)	19.239 (20)	15.279 (20)	22.070 (20)	16.964 (20)	24.922 (20)	17.433 (20)
MgO	32.929 (33)	30.489 (28)	32.782 (33)	30.807 (28)	29.353 (33)	30.631 (28)	26.362 (33)	31.035 (28)
SO_3_	0.063	1.721 (5)	0.061	4.541 (5)	0.036	2.993 (5)	0.078	3.004 (5)
Al_2_O_3_	0.648	0.858	0.724	0.872	0.802	0.685	1.121	1.088
Na_2_O	0.839	1.028	0.826	1.176	0.763	1.041	0.686	1.191
CaO	0.131	0.149	0.179	0.133	0.145	0.124	0.733	0.167
TiO_2_	0.016	0.022	0.021	0.024	0.036	0.030	0.000	0.033
Fe_2_O_3_	0.014	0.020	0.017	0.018	0.019	0.020	0.032	0.020
CuO	0.006	0.004	0.000	0.005	0.011	0.000	0.017	0.005
ZnO	0.004	0.002	0.000	0.000	0.003	0.000	0.000	0.002
Rb_2_O	0.070	0.012	0.020	0.010	0.025	0.011	0.040	0.013
SrO	0.003	0.002	0.002	0.002	0.003	0.000	0.005	0.003
ZrO_2_	0.006	0.004	0.003	0.003	0.038	0.000	0.006	0.026
Cr_2_O_3_	0.000	0.000	0.005	0.000	0.000	0.000	0.000	0.000
NiO	0.000	0.000	0.005	0.000	0.000	0.000	0.000	0.000
Sm_2_O_3_	0.000	0.000	0.003	0.000	0.000	0.000	0.000	0.000
PtO_2_	0.000	0.000	0.000	0.000	0.003	0.000	0.000	0.000
Cl	0.015	0.026	0.027	0.024	0.045	0.023	0.075	0.028

**Table 2:** The average true density value (*d_r_*) with its standard deviation (SD), molar volume, and optical basicity (Λ_th_) for the glass samples and glassy crystalline 36Si11P material.

	Glass Name	*d_r_* ± SD (g/cm^3^)	*V_mol_* (cm^3^/mol)	Λ_th_
Pure glass	45Si2P	2.5918 ± 0.0008	23.8145	0.6510
	43Si4P	2.5482 ± 0.0009	24.9547	0.6314
	39Si8P	2.5084 ± 0.0017	27.4620	0.6096
	36Si11P	2.5639 ± 0.0010	28.4816	0.5991
Sulfur doped glass	45Si2P5S	2.5682 ± 0.0010	23.5923	0.6229
	43Si4P5S	2.5433 ± 0.0008	23.7021	0.6051
	39Si8P5S	2.4919 ± 0.0028	25.3021	0.6020
	36Si11P5S	2.5381 ± 0.0009	25.9264	0.5859

**Table 3:** The positions of the peak centers of the deconvoluted MIR spectra of the pure and sulfur-doped silicate–phosphate glass samples in the particular ranges of wavenumbers, as well as the assignments of the bands to the appropriate vibrations.

Wavenumber Range/cm^−1^	Glass Name				Assignment
	45Si2P	39Si8P	45Si2P5S	39Si8P5S	
	Peak Position/cm^−1^				
400–650	440	445	440	444	(O-Si-O) bending vibrations [50,51,52] (O-P-O) symmetric bending vibrations [55] for sulfur-doped glass samples: no evidence of bands from polysulfides (S-S stretching vibrations) [53,54]
	514	530	510	532	(Si-O-Si) bending vibrations [56]
	553	567	544	568	(O-P-O) asymmetric bending vibrations [55]
	588	603	583	604	(O-P-O) asymmetric bending vibrations [56] for sulfur-doped glass samples: no evidence of bands from disulfides (S-S stretching) [54,55]
	625	643	619	642	(O-P-O) bending vibrations [57,58] for sulfur-doped glass samples: no evidence of bands from (SO_4_^2−^) asymmetric bending vibrations [53]
650–760	705	740	716	730	(Si-O-Si), (Si-O-P) symmetric stretching vibrations [50,51,52]
	740	766	751	759	(Si-O-Si), (Si-O-P) symmetric stretching vibrations [50,51,52], (Si-O 3-4BO) asymmetric stretching vibrations [56]
800–1600	814	838	828	836	(Si-O-Si) bending vibrations [58,59]
	849	869	863	868	(Si-O-2NBO) stretching vibrations [56,60]
	912	925	921	926	(Si-O-2NBO) symmetric stretching vibrations [56,60]
	1010	1019	1012	1019	(Si-O-1NBO) asymmetric stretching vibrations [56,60] (PO_4_^3-^) symmetric stretching vibrations [55]
	1117	1119	1114	1123	(Si-O-Si) stretching vibrations [50] (O-P-O) symmetric stretching vibrations [61] for sulfur doped glass samples: no evidence of bands from (SO_4_^2−^) asymmetric stretching vibrations [53]
	1255	1261	1258	1268	Si-O-Si stretching vibrations [58,62] O-P-O asymmetric stretching vibrations [61]
	1395	1416	1401	1432	P=O stretching vibrations [57,63]

**Table 4:** The values of the band-gap energy for the glass samples 36Si11P5S, 39Si8P5S, 45Si2P5S, and 45Si2P in the case of indirectly allowed transitions; *n* = 2.

*n*	45Si2P	36Si11P5S	39Si8P5S	45Si2P5S
2	4 eV	2.14 eV	2.03 eV	2.09 eV

## Data Availability

Data sharing is not applicable to this article.

## Sample Availability

Department of Ceramics and Refractories, Faculty of Materials Science and Ceramics, AGH University of Science and Technology, A. Mickiewicza 30, 30-059 Kraków, Poland.

## References

[1] Fleet M.E. (2005). XANES spectroscopy of sulfur in earth materials. Can. Mineral..

[2] Wells A.F. (1986). Structural Inorganic Chemistry.

[3] Tsujimura T., Xue X., Kanzaki M., Walter M.J. (2004). Sulfur speciation and network structural changes in sodium silicate glasses: Constraints from NMR and Raman spectroscopy. Geochim. Cosmochim. Acta..

[4] Paris E., Giuli G., Carroll M.R., Davoli I. (2001). The valence and speciation of sulfur in glasses by X-ray absorption spectroscopy. Can. Mineral..

[5] Jugo P.J., Wilke M., Botcharnikov R.E. (2010). Sulfur K-edge XANES analysis of natural and synthetic basaltic glasses: Implications for S speciation and S content as function of oxygen fugacity. Geochim. Cosmochim. Acta.

[6] McKeown D.A., Muller I.S., Gan H., Pegg I.L., Stolte W.C. (2004). Determination of sulfur environments in borosilicate waste glasses using X-ray absorption near-edge spectroscopy. J. Non-Cryst. Solids.

[7] Hirashima H., Yoshida T., Brückner R. (1988). Redox equilibria and constitution of polyvalent ions in oxide melts and glasses, Glastechn. Ber. Glass Sci. Technol..

[8] Bingham P.A., Connelly A.J., Hand R.J., Hyatt N.C., Northrup P.A., Alonso Mori R., Glatzer P., Kavčič M., Žitnik M., Bučar K. (2010). A multi-spectroscopic investigation of sulfur speciation in silicate glasses and slags. Glass Technol. Eur. J. Glass Sci. Technol. A.

[9] Angell C.A. (1965). Sulfate and sulfate-chloride glasses. J. Am. Ceram. Soc..

[10] Ojovan M.I., Lee W.E. (2005). An Introduction to Nuclear Waste Immobilization.

[11] Ghosh K., DasMohapatra G.K., Soodbiswas N. (2003). Glass formation in K_2_SO_4_-CaO-P_2_O_5_ system. Phys. Chem. Glasses.

[12] Malugani J.P., Mercier R., Fahys B., Robert G. (1982). Ionic conductivity of and Raman spectroscopy investigation in binary oxosalts (1 − x)AgPO_3_·xAg_2_SO_4_ glasses. J. Solid State Chem..

[13] Reibstein S., Da N., Simon J.P., Spiecker E., Wondraczek L. (2012). Phase separation and crystal precipitation in supercooled sulphophosphate ionic melts. Phys. Chem. Glas. Eur. J. Glas. Sci. Technol. Part B.

[14] Stefanovskii S.V., Aleksandrov A.I. (1991). EPR spectra and structure of sodium sulfate borate glasses. Zhurnal Prikladnoi Spektroskopii.

[15] Wincott P.L., Vaughan D.J. (2006). Spectroscopic Studies of Sulfides. Rev. Mineral. Geochem..

[16] Lane M.D. (2007). Mid-infrared emission spectroscopy of sulfate and sulfate-bearing minerals. Am. Mineral..

[17] Jaroudi O.E., Picquenard E., Demortier A., Lelieur J.-P., Corset J. (2000). Polysulfide anions II: Structure and vibrational spectra of the S_4_^2-^ and S_5_^2-^ anions. Influence of the cations on bond length, valence, and torsion angle. Inorg. Chem..

[18] Santagneli S.H., Schneider J., Skripachev I., Ribeiro S.J., Messaddeq Y. (2008). Preparation and characterization of new glassy system As_2_P_2_S_8_-Ga_2_S_3_. J. Phys. Chem. B.

[19] Kim Y., Saienga J., Martin S.W. (2005). Glass formation in and structural investigation of Li2S+GeS2+GeO2 composition using Raman and IR spectroscopy. J. Non-Cryst. Solids.

[20] Bischoff C., Schuller K., Dunlap N., Martin S.W. (2014). IR, Raman, and NMR Studies of the Short-Range Structures of 0.5Na2S + 0.5[xGeS_2_ + (1–x)PS_5/2_] Mixed Glass-Former Glasses. J. Phys. Chem. B.

[21] Le Q.H., Palenta T., Benzine O., Griebenow K., Limbach R., Kamitsos E.I., Wondraczek L. (2017). Formation, structure and properties of fluoro-sulfo-phosphate poly-anionic glasses. J. Non-Cryst. Solids.

[22] Kmiec S., Joyce A., Martin S.W. (2018). Glass formation and structural analysis of Na_4_P_2_S_7_-xO_x_, 0 ≤ x ≤ 7 sodium oxy-thiophosphate glasses. J. Non-Cryst. Solids.

[23] Thieme A., Möncke D., Limbach R., Fuhrmann S., Kamitsos E.I., Wondraczek L. (2015). Structure and properties of alkali and silver sulfophosphate glasses. J. Non-Cryst. Solids.

[24] Goel A., McCloy J.S., Fox K.M., Leslie C.J., Riley B.J., Rodriguez C.P., Schweiger M.J. (2012). Structural analysis of some sodium and alumina rich high-level nuclear waste glasses. J. Non-Cryst. Solids.

[25] Ehrt D. (2009). Photoluminescence in glasses and glass ceramics. IOP Conf. Ser. Mater. Sci. Eng..

[26] Gerasimova V.I., Rybaltovskii A.O., Chernov P.V., Spasskii D.A. (2003). Color Centers in Sulfur-Doped Silica Glasses: Spectroscopic Manifestations of an SO_2_ Interstitial Molecule. Glas. Phys. Chem..

[27] Gerasimova V.I., Zavorotny Y.S., Rybaltovskii A.O., Chernov P.V., Sazhin O.D., Khrapko R.R., Frolov A.A. (2002). Color Centers in Sulfur-Doped Silica Glasses: Spectroscopic Manifestations of an S^2+^ Interstitial Molecular Ion. Glass Phys. Chem..

[28] Gerasimova V.I., Rybaltovskii A.O., Chernov P.V., Zimmerer G. (2002). The Influence of Silica Glass Matrix on the Spectra of Interstitial Molecules S2. Glass Phys. Chem..

[29] Shi Y., Zhang P., Yang D., Wang Z. (2020). Synthesis, photoluminescence properties and sensing applications of luminescent sulfur nanodots. Chem. Commun..

[30] Su G., Liu C., Deng Z., Zhao X., Zhou X. (2017). Size-dependent photoluminescence of PbS QDs embedded in silicate glasses. Opt. Mater. Express.

[31] Han N., Liu C., Zhang J., Zhao X., Heo J., Jiang Y. (2014). Infrared photoluminescence from lead sulfide quantum dots in glasses enriched in sulfur. J. Non-Cryst. Solids.

[32] Lezal D., Pedlíıková J., Zavadil J., Kostka P., Poulain M. (2003). Preparation and characterization of sulfide, selenide and telluride glasses. J. Non-Cryst. Solids.

[33] Seki M., Hachiya K., Yoshida K. (2003). Photoluminescence and states in the bandgap of germanium sulfide glasses. J. Non-Cryst. Solids.

[34] Stoch L., Stoch Z., Wacławska I. (2003). Silicate Glass Fertilizer. Patent.

[35] Wacławska I., Szumera M. (2009). Reactivity of silicate-phosphate glasses in soil environment. J. Alloys Compd..

[36] Sułowska J., Wacławska I., Olejniczak Z. (2014). Effect of glass composition on the interactions between structural elements in Cu-containing silicate-phosphate glasses. J. Therm. Anal. Calorim..

[37] Nowotny W. (1958). Szkła Barwne.

[38] Manara D., Grandjean A., Pinet O., Dussossoy J.L., Neuville D.R. (2007). Sulfur behaviour in silicate glasses and melts: Implications for sulphate incorporation in nuclear waste glasses as a function of alkali cation and V_2_O_5_ content. J. Non-Cryst. Solids.

[39] Hassaan M.Y., El-Desoky M.M., Moustafa M.G., Iida L.Y., Kubuki S., Nishida T. (2015). Role of Sulfur as a Reducing Agent for the Transition Metals Incorporated into Lithium Silicate Glass. Croat. Chem. Acta.

[40] Shannon R.D. (1976). Revised effective ionic radii and systematic studies of interatomic distances in halides and chalcogenides. Acta Cryst..

[41] Baker L.L., Malcolm J. (1996). Rutherford, Sulfur diffusion in rhyolite melts. Contrib. Mineral. Petrol..

[42] Hassaan M.Y., Salem S.M., Moustafa M.G. (2014). Study of nanostructure and ionic conductivity of Li_1.3_Nb_0.3_V_1.7_(PO_4_)_3_ glass ceramics used as cathode material for solid batteries. J. Non-Cryst. Solids.

[43] El-Desoky M.M., Wally N.K., Sheha E., Kamal M. (2021). Impact of sodium oxide, sulfide, and fluoride-doped vanadium phosphate glasses on the thermoelectric power and electrical properties: Structure analysis and conduction mechanism. J. Mater. Sci. Mater. Electron..

[44] Xu X., Youngman R.E., Kapoor S., Goel A. (2021). Structural drivers controlling sulfur solubility in alkali aluminoborosilicate glasses. J. Am. Ceram. Soc..

[45] Kjeldsen J., Smedskjaer M.M., Mauro J.C., Youngman R.E., Huang L., Yue Y. (2013). Mixed alkaline earth effect in sodium aluminosilicate glasses. J. Non-Cryst. Solids.

[46] Shareefuddin M., Ramadevudu G., Rao S., Narasimha Chary M. (2013). Physical, Optical, and Spectroscopic Studies on MgO-BaO-B_2_O_3_ Glasses. Int. Sch. Res. Not..

[47] Sastry S.S., Rao B.R.V. (2014). Structural and optical properties of vanadium doped alkaline earth lead zinc phosphate glasses. Indian J. Pure Appl. Phys..

[48] Dimitrov V., Komatsu T. (2010). An interpretation of optical properties of oxides and oxide glasses in terms of the electronic ion polarizability and average single bond strength. J. Univ. Chem. Technol. Metall..

[49] Yousef E.S., Elokr M.M., AbouDeif Y.N. (2016). Optical, elastic properties and DTA of TNZP host tellurite glasses doped with Er3+ ions. J. Mol. Struct..

[50] Sitarz M. (2008). Influence of modifying cations on the structure and texture of silicate–phosphate glasses. J. Mol. Struct..

[51] Sitarz M. (2011). Structure of simple silicate glasses in the light of Middle Infrared spectroscopy studies. J. Non-Cryst. Solids.

[52] Handke M., Sitarz M., Rokita M., Galuskin E.W. (2003). Vibrational spectra of phosphosilicate biomaterials. J. Mol. Struct..

[53] Coates J. (2006). Interpretation of infrared spectra, a practical approach. Encyclopedia of Analytical Chemistry.

[54] Trofimov B.A., Sinegovskaya L.M., Gusarova N.K. (2009). Vibrations of the S–S bond in elemental sulfur and organic polysulfides: A structural guide. J. Sulphur Chem..

[55] Miladi L., Oueslati A., Guidara K. (2016). Phase transition, conduction mechanism and modulus study of KMgPO_4_ compound. RSC Adv..

[56] Lebecq I., Désanglois F., Leriche A., Follet-Houttemane C. (2007). Compositional dependence on the in vitro bioactivity of invert or conventional bioglasses in the Si-Ca-Na-P system. J. Biomed. Mater. Res. A.

[57] Chakraborty I.N., Condrate R.A. (1985). The Vibrational Spectra of Glasses in the Na_2_O-SiO_2_-P_2_O_5_ System with a 1:1 SiO_2_:P_2_O_5_ Molar Ratio. Phys. Chem. Glasses.

[58] Serra J., González P., Liste S., Serra C., Chiussi S., León B., Pérez-Amor M., Ylänem H.O., Hupa M. (2003). FTIR and XPS studies of bioactive silica based glasses. J. Non-Cryst. Solids.

[59] McKeown D.A., Bell M.I., Kim C.C. (1993). Raman spectroscopy of silicate rings: Benitoite and the three-membered ring. Phys. Rev. B.

[60] Aguiar H., Serra J., González P., León B. (2009). Structural study of sol–gel silicate glasses by IR and Raman spectroscopies. J. Non-Cryst. Solids.

[61] Gao H., Tan T., Wang D. (2004). Effect of composition on the release kinetics of phosphate controlled release glasses in aqueous medium. J. Control. Release.

[62] Villegas M.A., Navarro J.M.F. (1998). Characterization of B_2_O_3_-SiO_2_ glasses prepared via sol-gel. J. Mater. Sci..

[63] Ahsan M.R., Mortuza M.G. (2005). Infrared spectra of xCaO(1-x-z)SiO_2_zP_2_O_5_ glasses. J. Non-Cryst. Solids.

[64] Szumera M., Wacławska I. (2007). Spectroscopic and thermal studies of silicate-phosphate glasses. J. Therm. Anal. Calorim..

[65] Varshneya A.K. (1993). Fundamentals of Inorganic Glasses.

[66] Sułowska J., Jeleń P., Olejniczak Z., Szumera M. (2021). Sulfur speciation and network structural changes in silicate-phosphate glasses. J. Non-Cryst. Solids.

[67] Kęcki Z. (1992). Podstawy Spektroskopii Molekularnej.

[68] Tauc J. (1968). Optical properties and electronic structure of amorphous Ge and Si. Mater. Res. Bull..

[69] Wu X.L. (2001). Photoluminescence and cathodoluminescence studies of stoichiometric and oxygen-deficient ZnO films. Appl. Phys. Lett..

[70] Skuja L., Hosono H., Hirano M. (2001). Laser-induced color centers in silica. Laser-Induced Damage in Optical Materials: 2000.

[71] Garcia-Guinea L., Correcher V., Sanchez-Muñoz L., Finch A.A., Hole D.E., Townsend P.D. (2007). On the luminescence emission band at 340 nm of stressed tectosilicate lattices. Nucl. Instrum. Methods Phys. Res. A Accel. Spectrom. Detect. Assoc. Equip..

[72] Fredholm Y.C., Karpukhina N., Law R.V., Hill R.G. (2010). Strontium containing bioactive glasses: Glass structure and physical properties. J. Non-Cryst. Solids.

[73] Kasymdzhanov M.A., Khabibullaev P.K., Kurbanov S., Kurbanov E. (2001). UV-laser coloration and bleaching of unirradiated and gamma-irradiated silica glasses. Laser-Induced Damage in Optical Materials: 2000.

